# Spatial and Functional Crosstalk between the Mitochondrial Na^+^-Ca^2+^ Exchanger NCLX and the Sarcoplasmic Reticulum Ca^2+^ Pump SERCA in Cardiomyocytes

**DOI:** 10.3390/ijms23147948

**Published:** 2022-07-19

**Authors:** Ayako Takeuchi, Satoshi Matsuoka

**Affiliations:** 1 Department of Integrative and Systems Physiology, Faculty of Medical Sciences, University of Fukui, Fukui 910-1193, Japan; 2 Life Science Innovation Center, University of Fukui, Fukui 910-1193, Japan

**Keywords:** mitochondria, sarcoplasmic reticulum, NCLX, SERCA, Ca^2+^ signaling, cardiomyocyte

## Abstract

The mitochondrial Na^+^-Ca^2+^ exchanger, NCLX, was reported to supply Ca^2+^ to sarcoplasmic reticulum (SR)/endoplasmic reticulum, thereby modulating various cellular functions such as the rhythmicity of cardiomyocytes, and cellular Ca^2+^ signaling upon antigen receptor stimulation and chemotaxis in B lymphocytes; however, there is little information on the spatial relationships of NCLX with SR Ca^2+^ handling proteins, and their physiological impact. Here we examined the issue, focusing on the interaction of NCLX with an SR Ca^2+^ pump SERCA in cardiomyocytes. A bimolecular fluorescence complementation assay using HEK293 cells revealed that the exogenously expressed NCLX was localized in close proximity to four exogenously expressed SERCA isoforms. Immunofluorescence analyses of isolated ventricular myocytes showed that the NCLX was localized to the edges of the mitochondria, forming a striped pattern. The co-localization coefficients in the super-resolution images were higher for NCLX–SERCA2, than for NCLX–ryanodine receptor and NCLX–Na^+^/K^+^ ATPase α-1 subunit, confirming the close localization of endogenous NCLX and SERCA2 in cardiomyocytes. The mathematical model implemented with the spatial and functional coupling of NCLX and SERCA well reproduced the NCLX inhibition-mediated modulations of SR Ca^2+^ reuptake in HL-1 cardiomyocytes. Taken together, these results indicated that NCLX and SERCA are spatially and functionally coupled in cardiomyocytes.

## 1. Introduction

The heart possesses huge amounts of mitochondria which serve to supply ATP via oxidative phosphorylation, meeting the cardiac energy demand. The mitochondrial Ca^2+^—one of the key factors of mitochondrial energetics—is strictly maintained within an appropriate range, but once the maintenance is disrupted, cardiomyocytes can no longer function properly. An insufficient mitochondrial Ca^2+^ may disable the adequate energy production by the mitochondria, whereas an excess of mitochondrial Ca^2+^ opens mitochondrial permeability transition pores, causing cell death (see reviews [1,2]).

The mitochondrial Ca^2+^ in cardiomyocytes is balanced by an influx via the Ca^2+^ uniport (CU_mit_) activity and by an efflux via the Na^+^-Ca^2+^ exchange (NCX_mit_) or the H^+^-Ca^2+^ exchange (HCX_mit_) activities with the former plays the major part [3,4] (see also reviews [5,6,7]). It has been more than a decade since the molecules responsible for the activities were identified—MCU and its accessory proteins such as MICUs, EMRE, and so on for CU_mit_, NCLX for NCX_mit_, and possibly Letm1 for HCX_mit_—and the physiological/pathophysiological roles of these proteins in the heart have been extensively studied [8,9,10,11,12].

In ventricular myocytes, the mitochondria are densely and regularly arranged. According to the spatial distributions, they are divided into three groups—subsarcolemmal mitochondria (SSM) just beneath the sarcolemma, interfibrillar mitochondria (IFM) between the myofibrils, and perinuclear mitochondria (PNM) near the nucleus—and the heterogeneous distribution may contribute to dealing with region-specific cellular functions, such as region-specific energy demands and Ca^2+^ signaling [13,14]. A recent report showed that CU_mit_ activity was higher at the IFN than at the PNM, while the Ca^2+^ efflux activity was comparable between the two [15]. Another report proposed a spatial and functional coupling of NCLX with the voltage dependent Na^+^ channel Na_v_1.5, which was prominent at the SSM but not at the IFM [16]. In addition to intracellular heterogeneity, the intra-mitochondrial heterogeneity of the Ca^2+^ dynamics was also suggested. De La Fuente et al. [17,18] reported that protein expressions of MCU and EMRE, and activity of CU_mit_ were higher at mitochondria facing the junctional sarcoplasmic reticulum (jSR), where the sarcoplasmic reticulum (SR) Ca^2+^ release channel ryanodine receptor (RyR) 2 exists, than at the Percoll-purified mitochondria, whereas the protein expression of NCLX and activity of NCX_mit_ were higher at the Percoll-purified mitochondria than those at the jSR-attached mitochondria. Interestingly, our group found that an NCLX reduction decreased the SR/endoplasmic reticulum (ER) Ca^2+^ content, which might be attributable to the diminished activity of the SR/ER Ca^2+^ pump (SERCA) in HL-1 cardiomyocytes, in murine sinoatrial node cells, as well as in B lymphocytes [19,20,21]. These findings suggested the functional coupling of NCLX and SERCA, which might be effective if both proteins were closely localized with each other. The functional coupling of NCLX and SERCA is in line with the concept by De La Fuente et al. [18], namely, that spatial separation of MCU-RyR2 around the jSR from NCLX contributes to preventing the short-circuit of mitochondrial Ca^2+^ signaling; however, there is no information to date on the spatial relationships of NCLX with SERCA and on their physiological impact. In the present study, we explored the regional distribution of NCLX, especially focusing on the coupling with SERCA, in cardiomyocytes. We uncovered that NCLX and SERCA were localized in close proximity to each other, and that the biased localization of mitochondrial and SR Ca^2+^ channels/transporters contributes to efficient Ca^2+^ cycling between the mitochondria and SR in cardiomyocytes.

## 2. Results

### 2.1. Exogenously Expressed NCLX Was Closely Localized with SERCA in HEK293 Cells

**Figure 1:**
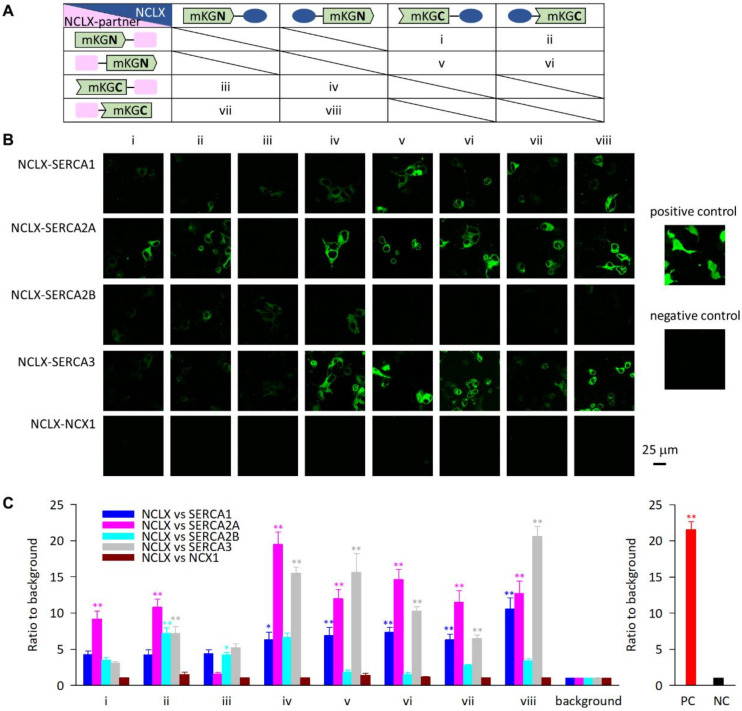
Bimolecular fluorescence complementation assay for NCLX and NCLX-partner proteins—four isoforms of SERCA and NCX1—in HEK293 cells. (**A**) The complementary pairs of mKGN- and mKGC-fused NCLX and NCLX-partner proteins at N- or C-terminus are numbered as i–viii. (**B**) Representative images of i–viii complementary pairs for combinations NCLX–SERCA1, NCLX–SERCA2A, NCLX–SERCA3, and NCLX–NCX1. As a positive and a negative control, representative images of a mKGN-p65 and mKGC-p50 combination and a mKGN fused with NCLX N-terminus only are shown in the right, respectively. (**C**) Fluorescence intensity/area of positive cells, which were normalized with those of background cells. For combinations of NCLX and NCLX-partner proteins, data are expressed as mean ± s.e.m. of 8–37 cells. * *p* < 0.05, ** *p* < 0.01, compared with background. For positive control (PC) and negative control (NC), data are expressed as mean ± s.e.m. of 138 and 140 cells, respectively. ** *p* < 0.01, compared with negative control.

First, we studied the molecular interactions of exogenously expressed NCLX with four isoforms of SERCA—SERCA1, SERCA2A, SERCA2B, and SERCA3—in HEK293 cells. SERCA1 and SERCA2A are expressed predominantly in fast-twitch skeletal muscle and cardiac muscle, respectively, whereas SERCA2B and SERCA3 are ubiquitously expressed in broad types of tissues (see review [22]). To test the interactions, we performed a bimolecular fluorescence complementation assay and evaluated the fluorescence intensity emitted from reconstructed steric monomeric Kusabira-Green (mKG) by co-expressing NCLX and SERCA expression plasmids fused with complementary pairs of a N- or C-terminal fragment of mKG (mKGN or mKGC) (see pairs i–viii in Figure 1A). As clearly shown in Figure 1B,C, at least two pairs in all the NCLX–SERCA combinations presented positive signals. As a reference, we chose a plasma membrane Na^+^-Ca^2+^ exchanger (NCX) 1, in which both the N- and C-terminus are facing extracellular space, confirming that no positive signals were obtained in any pairs of the NCLX–NCX1 combination (Figure 1B,C). It should be noted that SERCA2B possesses 11 transmembrane domains (TMs) and only the N-terminus is facing cytoplasmic space [23], while the other three isoforms have 10 TMs with both an N- and C-terminus facing cytoplasmic space; therefore, truncated mKG fused with the C-terminus of SERCA2B should not interact with the other truncated half of mKG. In fact, NCLX–SERCA2B co-transfection pairs corresponding to pairs v–viii in the Figure 1A did not show any positive signals (see the light blue bars in Figure 1C). Regarding NCLX, the topology and the three-dimensional (3D) structure have not been clarified yet; however, a recently developed artificial intelligence (AI)-based structure prediction method, AlphaFold [24], visualized that the C-terminus of NCLX is facing the opposite side of the long regulatory loop that is hypothesized to face the matrix, while the N-terminus contains a putative mitochondria transit peptide, the destiny of which is still unknown (see pdb file AF-Q6J4K2-F1-model_v2 in AlphaFold Protein Structure Database, https://alphafold.ebi.ac.uk/ (accessed on 13 December 2021) and review [6]). From this prediction, it is strongly suggested that at least the C-terminus of NCLX is accessible to cytoplasmic space, making the fused truncated mKG—found in pairs ii, iv, vi, and viii in Figure 1A—possible to react with the complement half of the mKG fused with SERCAs. Consistent with the idea, pairs ii, iv, vi, and viii of the NCLX–SERCA2A and NCLX–SERCA3 combinations, pairs iv, vi, and viii of the NCLX–SERCA1 combination, and pair ii of the NCLX–SERCA2B combination presented positive signals (Figure 1), suggesting that the C-terminus of the NCLX and the N- or C-terminus of the SERCAs were localized close together in the HEK293 cells. Though several pairs containing an N-terminus of NCLX fused with the truncated mKG also showed positive signals, we suspended their interpretation because the endogenous processing of the N-terminus of NCLX remains under study. Accordingly, it was suggested that exogenously expressed NCLX was closely localized with SERCAs.

### 2.2. Endogenous NCLX at Cardiac Mitochondria Was Restricted to the Area Where SR Were Attached

**Figure 2:**
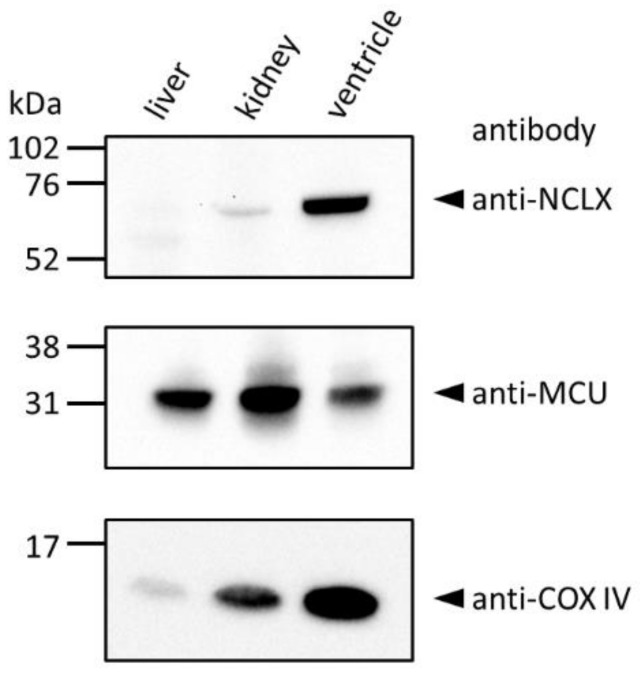
Western blot analyses of mouse liver, kidney and ventricular mitochondria. Isolated mitochondria (25 μg) were used for the experiments. The identity of mitochondria was confirmed by using anti-MCU and anti-COX IV antibodies.

Western blot analyses of isolated mitochondria using our custom-made anti-NCLX antibody [25] showed a strong single band especially in the ventricle (Figure 2), comparable with functional data showing that NCX_mit_ activity was high in excitable tissues such as the heart [3,26,27,28]. The band was diminished by pre-incubating the antibody with an excess amount of antigen peptide, confirming the quality of the antibody (Figure S1). Only faint signals were observed in liver and kidney mitochondria (Figure 2), again comparable with the functional data showing that NCX_mit_ activity was low in non-excitable tissues such as the liver and kidney [28,29].

**Figure 3:**
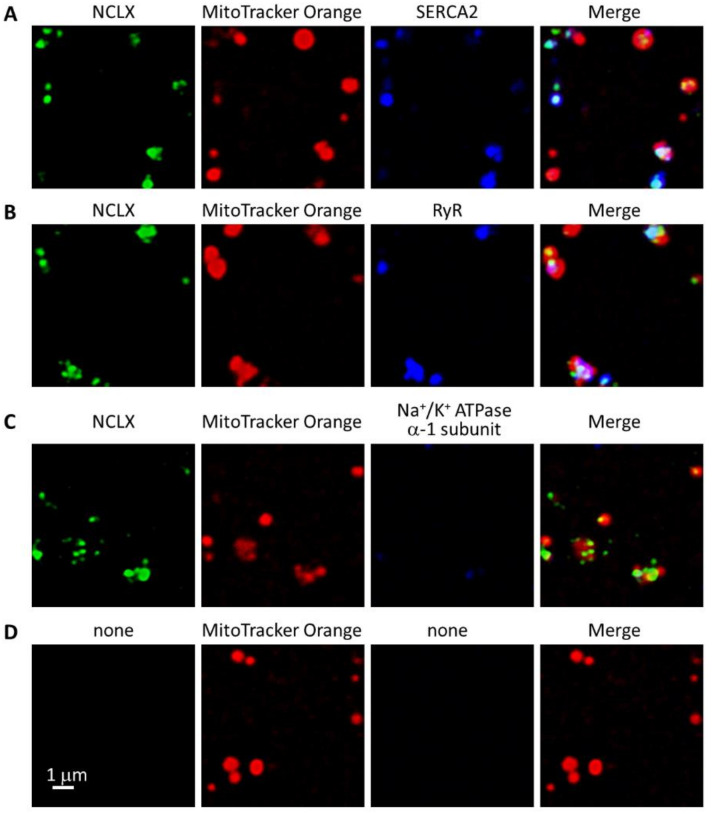
Immunofluorescence analyses of mitochondria isolated from mouse ventricle. Mitochondria loaded with 1 μM MitoTracker Orange CMTMRos were co-immunostained with anti-NCLX antibody and anti-SERCA2 (**A**), anti-RyR (**B**), and anti-Na^+^/K^+^ ATPase α-1 subunit (**C**) antibodies. NCLX-derived Alexa Fluor 488 signals are shown as green, MitoTracker Orange CMTMRos-derived signals are red, and SERCA2-, RyR-, and Na^+^/K^+^ ATPase α-1 subunit-derived Alexa Fluor 633 signals are blue. Merged images are shown in the rightmost panels. As a negative control, 1 μM MitoTracker Orange CMTMRos-loaded mitochondria, which was incubated with secondary antibodies but not with primary antibody, are shown in (**D**).

We previously performed immunofluorescence analyses using isolated brain mitochondria and found that NCLX was restrictedly localized to a portion of the mitochondria [25]. Considering that the isolated brain mitochondria contained some amounts of ER [25], we hypothesized that SR might be attached to the NCLX-localized area on cardiac mitochondria. Immunofluorescence analyses using isolated cardiac mitochondria showed that the signals derived from NCLX were restricted to a portion of the MitoTracker Orange CMTMRos positive area, where strong signals derived from SERCA2 and RyR were also observed, supporting the hypothesis (Figure 3A,B). Signals derived from a plasma membrane marker Na^+^/K^+^ ATPase α-1 subunit were negligible in the preparations (Figure 3C).

### 2.3. NCLX Was Localized to the Edges of Mitochondria in Adult Mouse Ventricular Myocytes

Next, we evaluated the spatial distribution of NCLX in adult mouse ventricular myocytes. The quality of our custom-made anti-NCLX antibody for immunofluorescence analyses of cardiomyocytes was tested using a HL-1 cell line, which was originated from mouse atrial myocytes. Unlike isolated adult ventricular myocytes, HL-1 has an advantage in that it can easily be genetically manipulated, though the efficacy is not high [20]. An NCLX knockdown using four different kinds of NCLX siRNA caused 20–40% reductions of the NCLX mRNA expressions (Figure S2A). Immunofluorescence analyses of HL-1 cells showed that most of the NCLX-derived signals were overlapped with the mitochondria-targeted pTagRFP-mito signals, though a part of the NCLX-derived signals appeared to exist outside the pTagRFP-mito signals, suggesting additional extra-mitochondrial localization of the NCLX (Figure S2B). NCLX knockdown significantly diminished the NCLX-derived signals in pTagRFP-mito-positive cells compared with those in cells transfected with negative control siRNA (Figure S2B,C). The histograms of the NCLX-derived signal intensities from the NCLX knockdown cells had left-shifted peaks compared with that from the negative control siRNA-transfected cells (Figure S2D). It should be noted that the NCLX-derived signal was almost completely diminished in some NCLX siRNA-transfected cells. Moreover, cells transfected with mouse NCLX-pCMV6 showed positive signals under the condition of a lower excitation laser intensity so that endogenous NCLX-derived signals were undetectable (Figure S2E), further confirming the quality of the antibody.

**Figure 4:**
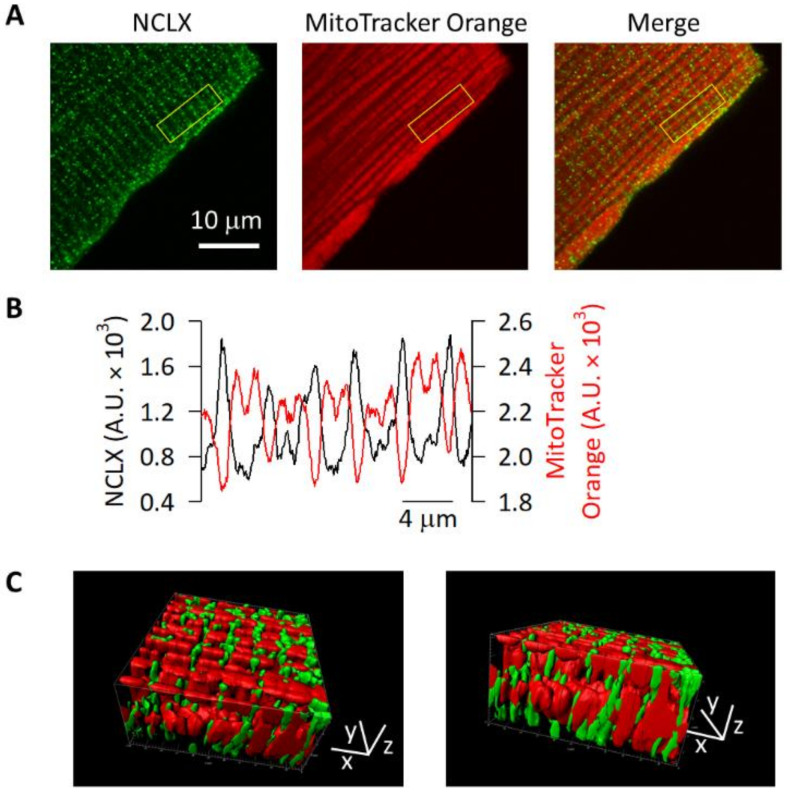
Immunofluorescence of NCLX in mouse ventricular myocytes. Isolated cells loaded with 1 μM MitoTracker Orange CMTMRos were immunostained with anti-NCLX antibody. (**A**) Images of a representative cell. NCLX-derived Alexa Fluor 488 signals and MitoTracker Orange CMTMRos-derived signals are shown as green (left) and as red (middle), respectively. The merged image is shown in the right. (**B**) Column average plots of the six sarcomeres-containing regions highlighted as yellow squares in (**A**). Profiles of NCLX- and MitoTracker Orange CMTMRos-derived signals are shown as black and red lines, respectively. (**C**) The 3D-reconstructed images of another representative cell with different angles. The rectangular region with 11 μm, 11 μm, and 5.2 μm for x, y, and z axis, respectively, is shown.

Immunofluorescence analyses of the adult mouse ventricular myocytes showed signals of a striped pattern with a transverse direction as well as signals along the sarcolemma, both of which were significantly decreased by pre-incubating the antibody with an excess amount of antigen peptide (Figure S3). The NCLX-derived signals along the sarcolemma were in good agreement with the report by Perez-Hernandez et al. [16], though stripe-like signals, especially in the middle of the myocytes, appeared much weaker in Perez-Hernandez et al. [16], possibly due to the poor penetration of the antibody that they used. In contrast, the analyses using the anti-MCU antibody showed no clear signals along the sarcolemma, while signals of a striped pattern were observed (Figure S4). When the cardiomyocytes were pre-loaded with MitoTracker Orange CMTMRos before the immunostaining, the signals derived from the NCLX were found between the signals derived from the MitoTracker Orange CMTMRos (Figure 4A). We focused on the NCLX-derived signals in the middle of the myocytes, that is to say, those at the IFM. The column average plot of the region containing six sarcomeres, highlighted as a yellow square in Figure 4A, showed alternately appearing fluorescence intensity peaks derived from the NCLX and MitoTracker Orange CMTMRos (Figure 4B). The 3D-reconstructed image revealed that the MitoTracker Orange CMTMRos area showed a rectangular pattern, which was surrounded by signals derived from the NCLX (Figure 4C). These results suggested that endogenous NCLX was localized to the edges of the mitochondria in the adult mouse ventricular myocytes.

### 2.4. NCLX Was Preferentially Localized near SERCA2 in Adult Mouse Ventricular Myocytes

**Figure 5:**
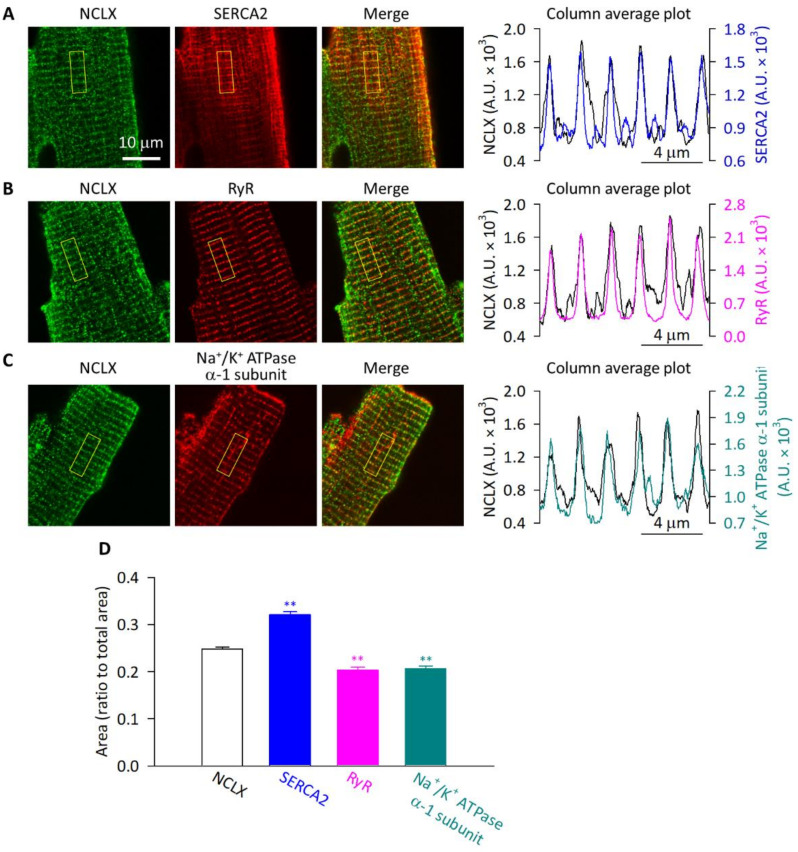
Immunofluorescence analyses of mouse ventricular myocytes. Isolated cells were co-immunostained with anti-NCLX antibody and anti-SERCA2 (**A**), anti-RyR (**B**), and anti-Na^+^/K^+^ ATPase α-1 subunit (**C**) antibodies, then images were obtained using a conventional confocal microscopy (Leica TCS SP II). NCLX-derived Alexa Fluor 488 signals and SERCA2-, RyR-, and Na^+^/K^+^ ATPase α-1 subunit-derived Alexa Fluor 633 signals are shown as green and as red, respectively. The merged images are also shown. Column average plots of the six sarcomeres-containing regions highlighted as yellow squares are shown in the rightmost panels. Profiles of NCLX are shown as black lines, and those of SERCA2, RyR, and Na^+^/K^+^ ATPase α-1 subunit are shown as dark blue, magenta, and cyan, respectively. (**D**) The signal-positive areas in the six sarcomeres-containing regions. Data are expressed as mean ± s.e.m. of 30–91 cells. ** *p* < 0.01, compared with NCLX.

Then we performed co-immunofluorescence analyses to assess the spatial relationships between the NCLX and SR- or sarcolemma-localized proteins in adult mouse ventricular myocytes. As shown in Figure 5A–C, the NCLX-derived striped pattern signals were close to the SERCA2-, RyR-, and Na^+^/K^+^ ATPase α-1 subunit-derived striped pattern signals. This was confirmed by the column average plots of the regions highlighted as yellow squares containing six sarcomeres—the periodic peaks of fluorescence intensity derived from the NCLX and those from the SERCA2, RyR, and Na^+^/K^+^ ATPase α-1 subunit, were synchronized. The signals derived from the anti-SERCA2 antibody were weak in some portions of the myocytes (Figure 5A), possibly due to the poor penetration of the antibody; therefore, we chose the regions where the signals were clearly observed for the analyses. The results obtained by the co-immunofluorescence analyses suggested that the NCLX-derived striped pattern signals arose from the regions close to T-tubules. It should be noted, however, that NCLX-derived signals were also found between the stripes, shown as small peaks between the large periodic peaks in the column average plots. In order to quantify the distribution pattern, we binarized the six sarcomeres-containing square region in the images and then calculated the signal-positive area. It was shown that the area was significantly smaller for the RyR and Na^+^/K^+^ ATPase α-1 subunit than that for the NCLX (Figure 5D), suggesting that the RyR and Na^+^/K^+^ ATPase α-1 subunit were restrictedly localized, whereas the NCLX was more diffusively distributed between the T-tubules. The signal-positive area for SERCA2 was even larger than that for the NCLX, reflecting its longitudinal as well as transversal distribution shown as grid-like signals, consistent with previous reports [30,31,32].

**Figure 6:**
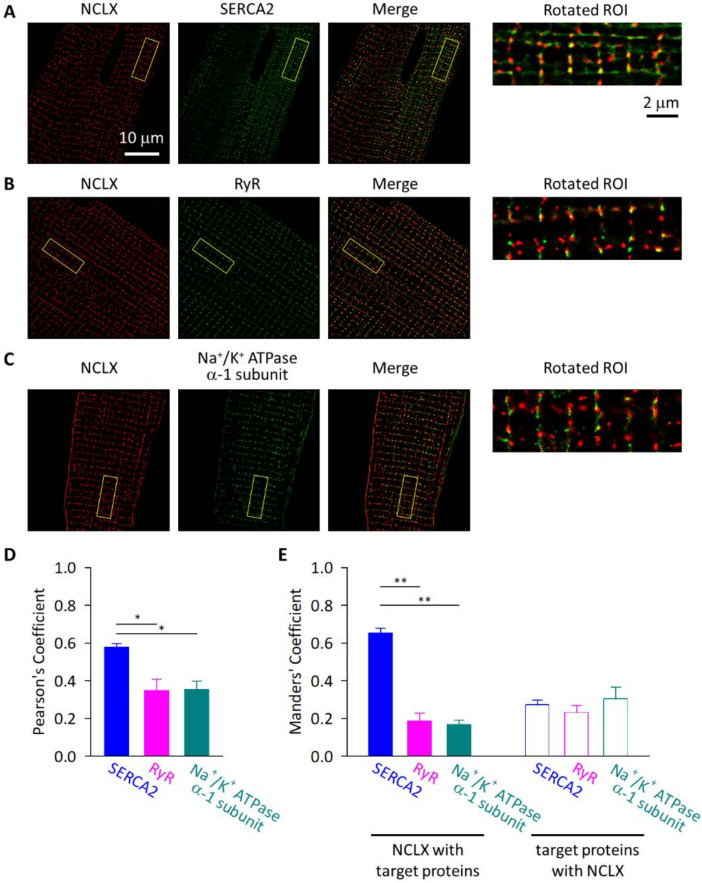
Super-resolution imaging of mouse ventricular myocytes. Isolated cells were co-immunostained with anti-NCLX antibody and anti-SERCA2 (**A**), anti-RyR (**B**), and anti-Na^+^/K^+^ ATPase α-1 subunit (**C**) antibodies, then images were obtained using an N-STORM (Nikon). NCLX-derived CF568 signals and SERCA2-, RyR-, and Na^+^/K^+^ ATPase α-1 subunit-derived Alexa Fluor 647 signals are shown as red and as green, respectively. The merged images are also shown. The ROIs for six sarcomeres-containing regions highlighted as yellow squares were rotated so that the longitudinal axis of the sarcolemma in the cardiomyocytes in each image became parallel to the x axis of the image (magnified images in the rightmost panels). (**D**) Pearson’s correlation coefficients for co-localization of NCLX and target proteins, SERCA2, RyR, and Na^+^/K^+^ ATPase α-1 subunit. (**E**) Manders’ co-localization coefficients for NCLX and target proteins. Fractions of NCLX co-localized with target proteins and those of target proteins co-localized with NCLX are shown as closed and open columns, respectively. Data are expressed as mean ± s.e.m. of 4–6 cells. * *p* < 0.05; ** *p* < 0.01.

We further evaluated the spatial distributions of the NCLX versus the SERCA2, RyR, and Na^+^/K^+^ ATPase α-1 subunit, by super-resolution imaging using a stochastic optical reconstruction microscopy (N-STORM) (Figure 6). Consistent with the images obtained by the conventional confocal microscopy, the signals derived from the NCLX showed a stripe-like pattern with an additional high intensity area in-between the stripes. The stripe-like part of the NCLX-derived signal was synchronized with the stripe-like signals derived from the SERCA2, RyR, and Na^+^/K^+^ ATPase α-1 subunit; however, magnified images revealed that the NCLX-derived signals and RyR- and Na^+^/K^+^ ATPase α-1 subunit-derived signals on the stripes did not much overlap, rather, they appeared alternately (the regions highlighted as yellow squares). On the other hand, a considerable portion of the NCLX-derived signals, both on the stripes and in-between the stripes, appeared close to the SERCA2-derived signals. Co-localization analyses of the region revealed that the Pearson’s coefficient was the highest for the NCLX–SERCA2 images (Figure 6D). In addition, the Manders’ coefficient for the signal from the NCLX overlapping with the signal from the co-immunostained target proteins was also the highest for the NCLX–SERCA2 images, while the Manders’ coefficient for the signal from the co-immunostained target proteins overlapping with the signal from the NCLX was comparable among the combinations (Figure 6E). Taken together, the imaging analyses demonstrated that the NCLX was highly localized at the mitochondrial membrane that was facing the SERCA2 on the SR of adult ventricular myocytes.

### 2.5. SR Ca^2+^ Dynamics Were Modulated by Inhibiting NCLX in HL-1 Cardiomyocytes

**Figure 7:**
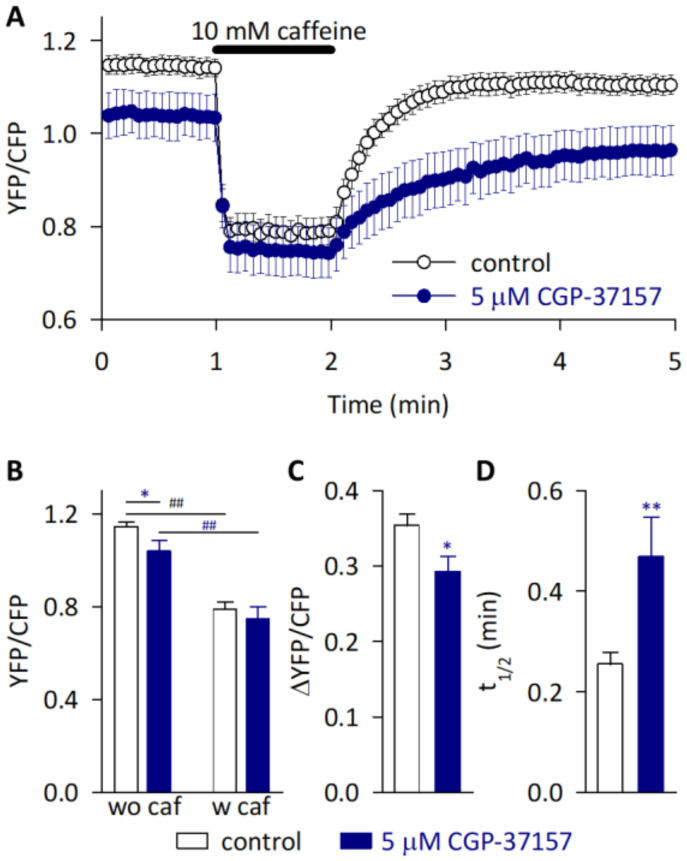
Effects of NCLX inhibition on the SR Ca^2+^ dynamics in HL-1 cardiomyocytes. (**A**) The SR Ca^2+^, evaluated as Cameleon D1ER-derived YFP/CFP, was emptied by applying 10 mM caffeine for 1 min, then the recovery was assessed for 3 min in the absence (control; white circles) or presence (blue circles) of an NCLX blocker, 5 μM CGP-37157. Data are expressed as mean ± s.e.m. of 8–12 cells. (**B**) SR Ca^2+^ level just before the application (wo caf) and in the presence (w caf) of 10 mM caffeine. * *p* < 0.05 vs. control, ^##^ *p* < 0.001 vs. wo caf. (**C**) SR Ca^2+^ content evaluated as ΔYFP/CFP. * *p* < 0.05 vs. control. (**D**) SR Ca^2+^ reuptake evaluated as half time of recovery, t_1/2_. ** *p* < 0.01 vs. control.

In order to get an insight into the functional consequences of the close localization of NCLX and SERCA, we investigated the contribution of NCLX to the SR Ca^2+^ dynamics of cardiomyocytes using a HL-1 cell line. HL-1 has high amounts of mitochondria and SR, which are intricately tangled with each other as in adult ventricular myocytes, while the T-tubules are less developed [5,20,33]. Taking the advantage that HL-1 cells can easily be genetically manipulated, we previously evaluated the SR Ca^2+^ dynamics in intact cells by expressing the SR/ER-specific Ca^2+^ sensor protein Cameleon D1ER, which was based on a ratiometric fluorescence resonance energy transfer (FRET) [34]. We found that the NCLX knockdown HL-1 cells from the use of siRNA had a reduced SR Ca^2+^ content and showed a slower reuptake of Ca^2+^ into emptied SR [20]. To exclude the contribution of compensated Ca^2+^ handlings caused by long-term NCLX reductions using siRNA, such as those caused by a physical distortion between mitochondria and the SR/ER as found in B lymphocytes [19], we pharmacologically inhibited NCLX by the use of 5 μM CGP-37157 (IC_50_ = 0.36 μM [35]) (Figure 7). The YFP/CFP fluorescence ratio derived from the Cameleon D1ER—an index of the SR Ca^2+^ level—before an application of 10 mM of caffeine was significantly smaller, while that ratio in the presence of caffeine was comparable, in the cells treated with 5 μM CGP-37157 (Figure 7A,B). This resulted in a smaller amount of YFP/CFP change (ΔYFP/CFP) by the caffeine treatment (Figure 7C), suggesting that the caffeine-responsive steady state SR Ca^2+^ content was reduced by inhibiting the NCLX. In addition, the recovery of the YFP/CFP fluorescence ratio after the caffeine removal was significantly slower in the presence of CGP-37157 (Figure 7A,D). These results were comparable to the previous observation using NCLX knockdown HL-1 cells [20]. Although a CGP-37157-mediated non-selective blockade of the L-type Ca^2+^ channel [36] might have affected the results, both molecular [20] and pharmacological (Figure 7) strategies indicated that a SERCA-mediated Ca^2+^ reuptake into the SR was diminished by the NCLX reduction/inhibition.

### 2.6. Functional Coupling between NCLX and SERCA as Revealed by Simulation Analyses

**Figure 8:**
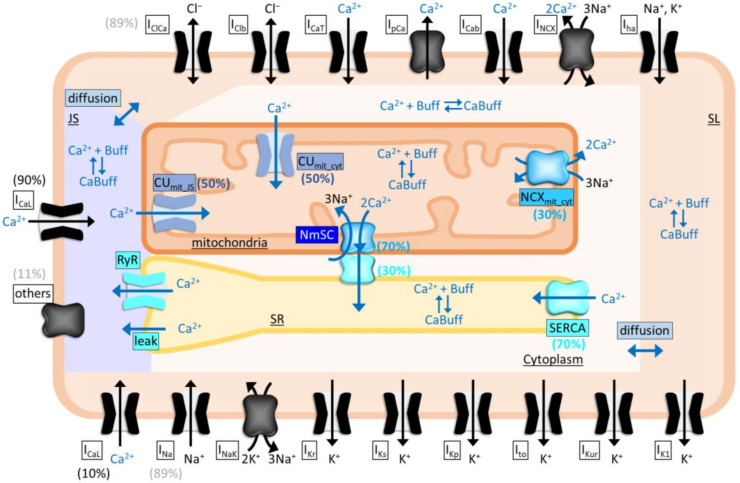
Schematic presentation of the mathematical model of HL-1 cardiomyocytes with mitochondria–SR interaction (MSI model). Distribution of L-type Ca^2+^ channel (I_CaL_) and other membrane channels/transporters (others) in junctional space (JS)/subsarcolemmal space (SL) are indicated in parenthesis with black and gray, respectively. Functional coupling of NCLX and SERCA is expressed by assuming that Ca^2+^, that is extruded through NCLX–SERCA complex (NmSC) from mitochondria, directly enters SR. Biased distributions of NmSC:NCX_mit_ facing cytoplasm (NCX_mit_cyt_) and NmSC:SERCA facing cytoplasm are set as 70%:30% and 30%:70%, according to the Manders’ coefficient of NCLX overlapping with SERCA2 and that of SERCA2 overlapping with NCLX as shown in Figure 6E, respectively. Distribution of CU_mit_ facing junctional space (CU_mit___JS_):CU_mit_ facing cytoplasm (CU_mit___cyt_) is set as 50%:50%, respectively, according to the super-resolution imaging of MCU by De La Fuente et al. [17].

We hypothesized that the NCLX-mediated modulation of SR Ca^2+^ dynamics were attributable to the spatial and functional coupling of NCLX and SERCA. To test the hypothesis, mathematical model analyses were performed. The HL-1 cell model [20] was updated so that the mitochondrial Ca^2+^ overload-mediated mitochondrial membrane depolarization and subsequent CU_mit_ inhibition were considered, by expressing them as simplified equations (see Supplementary Materials). The mitochondria–SR interaction (MSI) was implemented according to the experimental data on the biased distributions of NCLX, SERCA, and MCU (Figure 6 and [17])—in particular, the close-localization and functional coupling of NCLX and SERCA was expressed as “NCLX–SERCA complex (NmSC)”, through which Ca^2+^ extruded from the mitochondria directly enters the SR (MSI model; Figure 8).

**Figure 9:**
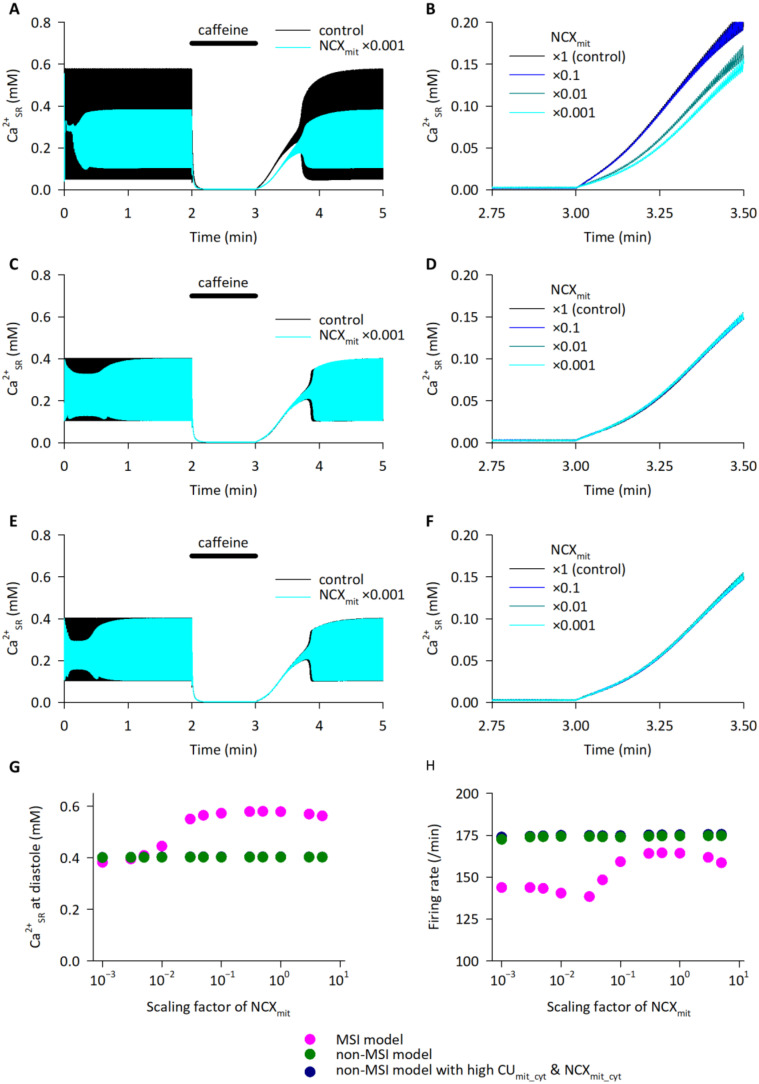
Model simulations of caffeine application and removal. (**A**) Time courses of SR Ca^2+^ concentrations obtained by using MSI model. Black and light blue lines represent simulated data with control and reduced scaling factor to ×0.001 of NCX_mit_ at time 0, respectively. (**B**) Same protocol was applied to the MSI model with various scaling factors of NCX_mit_, ×1 (control), ×0.1, ×0.01, and ×0.001. SR Ca^2+^ reuptake phase is magnified. (**C**) Time courses of SR Ca^2+^ concentrations obtained by using non-MSI model. Black and light blue lines represent simulated data with control and reduced scaling factor to ×0.001 of NCX_mit_ at time 0, respectively. (**D**) Same protocol was applied to the non-MSI model with various scaling factors of NCX_mit_, ×1 (control), ×0.1, ×0.01, and ×0.001. SR Ca^2+^ reuptake phase is magnified. (**E**) Time courses of SR Ca^2+^ concentrations obtained by using non-MSI model with high CU_mit_cyt_ and NCX_mit_cyt_. Black and light blue lines represent simulated data with control and reduced scaling factor to ×0.001 of NCX_mit_ at time 0, respectively. (**F**) Same protocol was applied to the non-MSI model with high CU_mit_cyt_ and NCX_mit_cyt_, with various scaling factors of NCX_mit_, ×1 (control), ×0.1, ×0.01, and ×0.001. SR Ca^2+^ reuptake phase is magnified. (**G**) Relationships between NCX_mit_ scaling factor and SR Ca^2+^ level at diastole just before the caffeine application. Magenta, green, and dark blue circles represent simulated data with MSI model, non-MSI model, and non-MSI model with high CU_mit_cyt_ and NCX_mit_cyt_, respectively. (**H**) Relationships between NCX_mit_ scaling factor and firing rate just before the caffeine application. Magenta, green and dark blue circles represent simulated data with MSI model, non-MSI model, and non-MSI model with high CU_mit_cyt_ and NCX_mit_cyt_, respectively.

The MSI model showed spontaneous generations of action potentials and cytosolic Ca^2+^ transients, which were comparable with those observed in the HL-1 cardiomyocytes [20] (magenta lines in Figure S5). In addition, the model well reproduced the experimental data on the 10 mM caffeine application shown in Figure 7—the caffeine-induced SR Ca^2+^ depletion and the caffeine removal-induced Ca^2+^ reuptake into the SR (black line in Figure 9A). It should be noted that the oscillations of SR Ca^2+^, shown in the simulations, were not detected in the experiments, possibly due to the low temporal resolution of the FRET imaging in the experiments.

The same caffeine application protocol was applied to the model with a reduced scaling factor of the NCX_mit_, mimicking a CGP-37157-mediated NCLX inhibition. The NCX_mit_ reduction in the MSI model reduced the steady state SR Ca^2+^ content and slowed the firing rate (Figure 9A,B and magenta circles in Figure 9G,H), well reproducing the experimental results. Moreover, the smaller the scaling factor of the NCX_mit_, the slower the SR Ca^2+^ reuptake rate after the caffeine removal became (Figure 9B), consistent with the experimental results. The slower SR Ca^2+^ reuptake rate was due to the diminished SR Ca^2+^ flux via the NmSC (Figure S6A).

A non-MSI model, in which the CU_mit_ facing junctional space (CU_mit_JS_) and NmSC were removed, also generated action potentials and cytosolic Ca^2+^ transients similar to the MSI model (green lines in Figure S5). Surprisingly, the NCX_mit_ reduction did not alter the SR Ca^2+^ level nor the SR Ca^2+^ reuptake rate (Figure 9C,D and green circles in Figure 9E), except for a transient decrease in the SR Ca^2+^ level within one min after the NCX_mit_ reduction (light blue line in Figure 9C). The SR Ca^2+^ flux via SERCA decreased only transiently by the NCX_mit_ reduction and returned to the similar level as the control level (Figure S6B). Accordingly, the firing rates were unaltered (green circles in Figure 9H).

To examine which factor is important for reproducing the experimental data, the biased distributions or the amplitudes of the scaling factors—i.e., expression levels, the non-MSI model with the increased activities in CU_mit_ facing cytoplasm (CU_mit___cyt_) and NCX_mit_ facing cytoplasm (NCX_mit_cyt_) (non-MSI model with high CU_mit_cyt_ and NCX_mit_cyt_) was constructed. The model failed to reproduce the experimental data on the effects of NCLX inhibition, indicating important roles of the biased distribution of CU_mit_ and NCX_mit_ (Figure 9E,F, and dark blue circles in the Figure 9G,H).

Taken together, the model analyses demonstrated that the biased distribution of mitochondrial and SR Ca^2+^ handling proteins and the functional coupling of NCLX and SERCA play pivotal roles in the SR Ca^2+^ dynamics and in the generation of automaticity in HL-1 cardiomyocytes.

## 3. Discussion

It was suggested that NCLX supplies Ca^2+^ from the mitochondria to the SR/ER in cardiomyocytes as well as in B lymphocytes [19,20,21]; however, mechanisms underlying the functional coupling between NCLX and SERCA were unknown. In the present study, we discovered that NCLX and SERCA are closely localized with each other, which is favorable for the efficient Ca^2+^ transfer from mitochondria to the SR in cardiomyocytes.

It is now generally accepted that the physical contacts between mitochondria and the SR/ER, called mitochondria-associated membranes (MAM), are crucial to Ca^2+^ signaling, reactive oxygen species production, autophagy, lipid metabolism, and so on, in various types of cells. Above all, Ca^2+^ transfer from the SR/ER to the mitochondria via the inositol-1,4,5 triphosphate receptor (IP_3_R) on the SR/ER, the voltage dependent anion channels (VDACs) on the outer membrane of mitochondria, and the CU_mit_ on the inner membrane of mitochondria, have been well studied (see reviews [37,38,39,40]). In the heart, the impairment of the Ca^2+^ transfer from the SR to the mitochondria via the IP_3_R–VDAC–CU_mit_ axis, which was observed in the mouse model of diabetic cardiomyopathy, disrupted the mitochondrial bioenergetics while the excitation–contraction coupling was unaffected [41]. De La Fuente et al. [17] reported another SR–mitochondria Ca^2+^ axis. They showed that the CU_mit_ components, MCU and EMRE, formed hotspots at the mitochondria–jSR association, which might contribute to locally receiving Ca^2+^ signals from RyR2, a major SR Ca^2+^ release channel governing the cardiac excitation–contraction coupling [17]. They also proposed that the CU_mit_ hotspots and NCLX were spatially separated to save on the mitochondrial energy cost, which was deduced from biochemical and functional assays using isolated organelle—crude mitochondria, Percoll-purified mitochondria, and jSR [18]. Here, we succeeded in visualizing the spatial separation between RyR and NCLX in intact ventricular myocytes, by immunofluorescence analyses using a conventional confocal microscopy as well as an N-STORM (Figure 5 and Figure 6), supporting the idea by De La Fuente et al. [18]. Moreover, our study is the first to demonstrate the reverse Ca^2+^ flow—from the mitochondria to the SR—via the spatial and functional coupling between NCLX and SERCA. Accordingly, these findings filled in the final piece in our understanding of the efficient Ca^2+^ cycling between the SR and mitochondria. The mathematical model analyses revealed that the experimentally observed efficient Ca^2+^ reuptake into the SR after the caffeine removal (Figure 7 and [20]) could not be reproduced without assuming the close proximity of NCLX and SERCA (Figure 9). It should be noted that the structural organization, such as T-tubule development, is different between HL-1 cells and adult ventricular myocytes, raising the possibility that the spatial separation between CU_mit_ hotspots and NCLX is less developed in HL-1 cells than in adult ventricular myocytes; therefore, the contribution of NCLX–SERCA coupling in adult ventricular myocytes might be even larger than that estimated in HL-1 cells. Further analyses specifically evaluating mitochondrial Ca^2+^ and SR Ca^2+^ in intact adult ventricular myocytes—though technically difficult to carry out at present—should give us more information to answer this question.

In our previous simulation study using a HL-1 cell model, which did not consider NCLX–SERCA coupling, a NCX_mit_ attenuation induced a reduction in the SR Ca^2+^ content and firing rate [20]. The reductions were caused mainly by Ca^2+^ accumulation in the mitochondria—i.e., the Ca^2+^ buffering effect of mitochondria. The mitochondrial Ca^2+^ buffering might have been overestimated in the previous model study, because we found that the simulation of a caffeine application protocol using the previous model resulted in a large increase in mitochondrial Ca^2+^ to an unphysiological level. In the present study, we simply assumed that high mitochondrial Ca^2+^ attenuates CU_mit_ to suppress an unphysiological mitochondrial Ca^2+^ overload during a caffeine application protocol. More quantitative experimental data, such as on mitochondrial Ca^2+^–ΔΨ depolarization–CU_mit_ activity relationships, are required for constructing a detailed mitochondrial model. In addition, another Ca^2+^ extruding system, HCX_mit_, was not considered in this mathematical model because the contribution of HCX_mit_ in the cardiomyocytes is believed to be small [12,42]; however, under the condition of suppressed NCX_mit_, it may play a significant role. To overcome the above limitation, further experimental and modelling studies remain to be performed.

There is a deviation between our findings and those by De La Fuente et al. [18]—the NCLX-derived immunofluorescence signals appeared near T-tubules with a stripe-like pattern as shown in the Figure 4, Figure 5 and Figure 6, whereas isolated jSR samples did not show an NCLX-derived positive band in Western blot analyses nor Na^+^-dependent, and CGP-37157-dependent Ca^2+^ efflux activity in the ^45^Ca^2+^ retention assay [18]. At present, we do not have a specific interpretation for the disparity, but different experimental approaches might have affected the results—e.g., the expression level of NCLX at mitochondria attached to the jSR in the samples of De La Fuente et al. [18] might have been too low to be detected by Western blot analyses. This well corresponds to the co-localization analyses of the N-STORM images (Figure 6E), where only a small fraction of NCLX (~0.2) was co-localized with the jSR-resident RyR. In addition, since some portions of the jSR-attached mitochondria were fragmented [17], they might have failed to maintain a suitable environment for NCLX activity, such as in the ion gradients and phosphorylation status. Accordingly, the NCLX activity in the jSR fraction might have been disturbed and been underestimated in the jSR fraction [18]. We cannot completely eliminate the possibility of non-specific bindings in the immunofluorescence signals, overestimating the distribution of NCLX near the T-tubules; however, the diminishment of NCLX-derived signals by transfection of the NCLX siRNAs in some—though not all due to the low transfection efficacy—HL-1 cells, assures the quality of the anti-NCLX antibody in detecting NCLX (Figure S2). Further analyses using cardiomyocytes isolated from conditional NCLX knockout mice [10] would clarify the point.

The topology of NCLX has not been clarified yet. In Kostic et al. [43], NCLX was suggested to possess one mitochondria transit peptide and 13 TMs whose C-terminus faced the same side of the long TM 5–6 loop. This topology model is consistent with the prediction by the SOSUI program (ver.1.11; https://harrier.nagahama-i-bio.ac.jp/sosui/mobile/ (accessed on 17 December 2021) [44]). On the other hand, it was predicted by the TopPred program [45] to possess one mitochondria transit peptide and 12 TMs whose C-terminus faced the opposite side of the long TM 5–6 loop, as presented in Cai and Lytton [46] and Kostic et al. [47]. A recently developed AI-based 3D structure prediction tool, AlphaFold [24], showed a similar prediction [6]. The results obtained by a bimolecular fluorescence complementation assay (Figure 1) support the latter prediction that the C-terminus of NCLX is facing the opposite side of the matrix. That is, in all the NCLX–SERCA combinations, at least one out of four pairs which contain NCLX C-terminally fused with truncated mKG, gave positive signals. Some pairs containing NCLX N-terminally fused with truncated mKG also gave positive signals, raising the possibility that the N-terminus of NCLX—containing the mitochondria transit peptide—is facing the anti-matrix side. Construction of mice harboring N-and C-terminally tagged NCLX would help with understanding the endogenous topology of NCLX; however, this is beyond the scope of the present study.

Considering that not only cardiac-specific isoform SERCA2A, but also other SERCA isoforms were shown to be closely localized with NCLX in the bimolecular fluorescence complementation assay (Figure 1), the Ca^2+^ transfer from the mitochondria to the SR/ER should also be operative in other types of cells. In fact, we previously found the modulation of ER Ca^2+^ handlings via NCLX in B lymphocytes, where SERCA2 and SERCA3 were expressed, contributing to the cytosolic Ca^2+^ response to an antigen receptor stimulation and chemotaxis [19,48] (see reviews [5,49]). The close proximity of NCLX and SERCA should have further promoted the mitochondria to ER Ca^2+^ transfer in these cells. Recent findings suggested a Ca^2+^ dysregulation in neurodegenerative diseases, such as Alzheimer’s disease and Parkinson’s disease (see reviews [50,51]). Interestingly, several research groups have reported the involvements of NCLX, SERCA, and MAM dysfunctions in the processes [52,53,54]. It would be interesting to examine the physical and functional coupling between NCLX and SERCA in neuronal cells and their roles in the pathogenesis of neurodegenerative diseases.

## 4. Materials and Methods

### 4.1. Bimolecular Fluorescence Complementation Assay

The HEK293 cell line (ATCC CRL-1573) was purchased from Summit Pharmaceuticals International and was maintained according to the manufacture’s protocol. The mouse open reading frame cDNA clones in pCMV6-Entry vector coding for SERCA1 (NM_007504), SERCA2A (NM_009722), SERCA2B (NM_001110140), and SERCA3 (NM_016745) were purchased from Origene Technologies. The mouse open reading frame cDNA clone coding for NCX1 (NM_011406) was a kind gift from Prof. Ottolia. The bimolecular fluorescence complementation assay was performed using a CoralHue Fluo-chase Kit (MBL), according to the manufacture’s protocol. In brief, the PCR-amplified cDNA fragments of mouse NCLX (NM_133221.2) [19], four SERCA isoforms, and NCX1 were inserted to the four types of vectors containing an N- or C-terminal fragment of mKG (mKGN or mKGC)—phmKGN-MC, phmKGC-MC, phmKGN-MN, and phmKGC-MN—to generate four types of recombinant fusion proteins for each clone. If mKGN and mKGC spatially approach each other—i.e., a protein fused with mKGN and a protein fused with mKGC localize nearby—a full mKG protein is reconstructed and a green fluorescence signal is generated. In order to detect the close localization of the two proteins, eight pairs of mKGN- and mKGC-fusion proteins were co-transfected into the HEK293 cells using Lipofectamine LTX Reagent (Thermo Fisher Scientific, Waltham, MA, USA) (i–viii pairs in Figure 1A). As a positive control, pCONT-1 and pCONT-2, containing p65 and p50 partial domains from NF-κB, respectively, were co-transfected. As a negative control, phmKGN-MC inserted with NCLX was used. Two days after the transfection, images were acquired using a laser scanning confocal microscope (Leica TCS SP II) with a ×63 oil objective lens (NA1.40). The excitation/emission wavelengths were 476/489–546 nm. The images were analyzed using Image J (NIH). The fluorescence intensity/area of each cell was corrected with the averaged fluorescence intensity/area of the background cells. For the images with no positive cells, ten cells were arbitrarily picked up according to the transmit images and used for the analyses.

### 4.2. Animals

All animal experimental procedures were approved by the Animal Research Committee, University of Fukui. Male 8–14 weeks old C57BL/6J mice (CLEA Japan, Inc., Shizuoka, Japan), which were housed in a 12 h light–dark cycle with ad libitum access to food and water, were used for the experiments.

### 4.3. Western Blot Analyses

The mice were heparinized (200 U/mouse, i.p.) and sacrificed by cervical dislocation. The liver, kidney and left ventricle were quickly excised after thoracotomy. Mitochondria were isolated by a differential centrifugation method and Western blot analyses were performed as described previously [25,27]. The primary antibodies used were: custom-made anti-NCLX (1:500, [25]), anti-MCU (1:1000, Cell Signaling Technology, Danvers, MA, USA, #14997), and anti-COX IV (1:2000, Abcam, Cambridge, UK, ab14744). The secondary antibodies used were: HRP-linked anti-rabbit IgG (Jackson ImmunoResearch, West Grove, PA, USA) for the NCLX and MCU, and HRP-linked anti-mouse IgG (GE healthcare, Chicago, IL, USA) for the COX IV. The quality of the custom-made anti-NCLX antibody, which was verified using HeLa cells transiently expressing NCLX and NCLX–FLAG [25], was further confirmed in Western blot analysis using cardiac mitochondria, where a single positive band around 65 kDa was diminished by pre-incubating the antibody with an excess amount of antigen peptide (Figure S1).

### 4.4. Immunofluorescence Analyses

Left ventricular myocytes were isolated by treating the adult mouse heart with collagenase using a Langendorff-type coronary perfusion [55], and stored in a KB buffer containing 70 mM K^+^ glutamate, 30 mM KCl, 10 mM KH_2_PO_4_, 1 mM MgCl_2_, 20 mM taurine, 0.3 mM EGTA, 10 mM glucose, and 10 mM HEPES (pH7.2 with KOH). Immunofluorescence analyses of isolated cardiac mitochondria and isolated ventricular myocytes which were pre-loaded with or without 1 μM MitoTracker Orange CMTMRos (Thermo Fisher Scientific, Waltham, WA, USA) were performed as described previously [25]. The primary antibodies used were: custom-made anti-NCLX (1:100, [25]), anti-MCU (1:100, Cell Signaling Technology, #14997), anti-SERCA2 (1:100, Santa Cruz Biotechnology, Inc., Dallas, TX, USA, sc-376235), anti-RyR (1:80, Thermo Fisher Scientific, MA3-916), and anti-Na^+^/K^+^ ATPase α-1 subunit (1:400, Merck, Darmstadt, Germany, 05-369). The secondary antibodies used for the confocal microscopic analyses were: anti-rabbit IgG Alexa Fluor 488 (1:500, Thermo Fisher Scientific) for the NCLX and MCU, and anti-mouse IgG Alexa Fluor 546 (1:500, Thermo Fisher Scientific) or anti-mouse IgG Alexa Fluor 633 (1:500, Thermo Fisher Scientific) for the SERCA2, RyR, and Na^+^/K^+^ ATPase α-1 subunit. The secondary antibodies used for the super-resolution microscopic analyses were: anti-rabbit IgG CF568 (1:500; Biotium, Fremont, CA, USA) for the NCLX and anti-mouse IgG Alexa Fluor 647 (1:500; Abcam) for the SERCA2, RyR, and Na^+^/K^+^ ATPase α-1 subunit.

For the analyses of isolated cardiac mitochondria, the images were acquired using a laser scanning confocal microscope (Olympus FV1200) with a ×100 oil objective lens (NA 1.35). The excitation/emission wavelengths were 473/485–545 nm, 559/570–625 nm, and 635/655–755 nm for the Alexa Fluor 488, MitoTracker Orange CMTMRos, and Alexa Fluor 633, respectively.

For the analyses of isolated ventricular myocytes, the images were acquired using a laser scanning confocal microscope (Leica TCS SP II) with a ×100 oil objective lens (NA1.40). The excitation/emission wavelengths were 488/502–524 nm, 543/555–651 nm, and 543/558–651 nm for the Alexa Fluor 488, MitoTracker Orange CMTMRos and Alexa Fluor 546, respectively. The quality of the custom-made anti-NCLX antibody in the immunofluorescence study was confirmed in NCLX knockdown HL-1 cells (Figure S2). It was also confirmed in mouse adult ventricular myocytes by pre-incubating the antibody with an excess amount of antigen peptide, which significantly diminished the fluorescence intensity (Figure S3). For the 3D reconstruction, Z-stack serial images with a 0.26 μm interval were deconvoluted using AutoQuant X3 (Media Cybernetics, Rockville, MD, USA) and then a 3D image was created using Imaris (Oxford Instruments, Abingdon, UK). For the quantification analyses, the region of interests (ROIs) of 11 μm width (six sarcomeres) and 3.67 μm height—i.e., 300 pixels × 100 pixels—in the middle of the myocytes were chosen. The column average plots were acquired using Image J. Distribution of the signals was analyzed using Image J—ROIs in the images were binarized and the signal-positive area was measured. The offset of each image was set automatically to avoid an arbitrary judgement. The ratio of the signal-positive area to the total ROI area (40.3 μm^2^) was calculated.

Super-resolution fluorescence imaging was performed using an N-STORM (Nikon, Tokyo, Japan) equipped with a ×100 oil objective lens (NA 1.49) and an ORCA-Flash 4.0 digital CMOS camera (Hamamatsu Photonics, Hamamatsu, Japan). In each acquisition run, 20,000 frames and 10,000 frames of 40 μm × 40 μm were recorded for the NCLX and for the SERCA2, RyR, and Na^+^/K^+^ ATPase α-1 subunit, respectively. The output of the activation 405 nm laser was set to 3%, and that of the imaging lasers of 561 nm and 647 nm were set to 100%. The data were processed to reconstruct the super-resolution images using NIS-Elements software (Nikon). For the co-localization analyses of the NCLX with various proteins, the ROIs with 12 μm × 4 μm—i.e., 1500 pixels × 500 pixels—in the middle of the myocytes were analyzed using the “Just Another Co-localization Plugin (JACoP)” of Image J. The offset of each image was set automatically to avoid an arbitrary judgement, then the Pearson’s correlation coefficients [56] and the Manders’ coefficients [57] were calculated.

### 4.5. Measurements of SR Ca^2+^ in HL-1 Cardiomyocytes

The HL-1 cell line was a kind gift from Dr. Claycomb [33] and was maintained according to the manufacture’s protocol as described previously [20]. A FRET-based SR Ca^2+^ indicator Cameleon D1ER/pcDNA3, which was a kind gift from Dr. Tsien [34], was transfected into the cells using Lipofectamine 3000 according to the manufacture’s protocol (Thermo Fisher Scientific). The experiments were performed 72 h after the transfection, as described previously, with slight modifications [20]. In brief, the cells were perfused with Tyrode’s solution containing 140 mM of NaCl, 5.4 mM KCl, 0.33 mM NaH_2_PO_4_, 0.5 mM MgCl_2_, 1.8 mM CaCl_2_, 5.5 mM glucose and 5 mM HEPES (pH 7.4 with NaOH). Then, 10 mM caffeine in Tyrode’s solution was applied for 1 min to empty the SR Ca^2+^ by switching the solution using a valve controlled gravity perfusion system (ALA Scientific Instruments, Farmingdale, NY, USA). The cells were excited at 435 ± 10 nm and fluorescence image pairs at 480 ± 30 (CGP) and 535 ± 40 nm (YFP), which were separated with a W-View system (Hamamatsu Photonics), were recorded every 4 sec using a cooled CCD digital camera (ORCA-ER, Hamamatsu Photonics). The images were analyzed using the AQUACOSMOS software (Hamamatsu Photonics). The YFP/CFP fluorescence intensity ratio of each cell was considered as the SR Ca^2+^ level of the cell, and the ΔYFP/CFP just before and during the caffeine application was considered as the SR Ca^2+^ content. The recovery rate of the YFP/CFP after the removal of caffeine was considered as the SR Ca^2+^ reuptake rate, which was evaluated by calculating the half time (t_1/2_) from a minimum YFP/CFP during the caffeine application to the steady-state value at 3 min after the removal of caffeine. In order to block NCLX, the experiments were performed in the presence of 5 μM CGP-37157.

### 4.6. Mathematical Model Analyses

A mathematical model of HL-1 cardiomyocytes considering MSI was developed based on our previous model [20]. The detailed description of the model is presented in the Supplementary Materials Tables S1–S8.

### 4.7. Statistical Analyses

The statistical analyses were performed by a Student’s *t*-test, paired *t*-test, and one-way ANOVA for unpaired two group comparisons, paired two group comparisons, and multiple comparisons, respectively (SigmaPlot 14.0). A *p*-value of <0.05 was determined as a statistically significant difference.

## Data Availability

Not applicable.

## Supplementary Materials

The following supporting information can be downloaded at: https://www.mdpi.com/article/10.3390/ijms23147948/s1.
