# Supplementary material for: Spatial and Functional Crosstalk between the Mitochondrial Na+-Ca2+ Exchanger NCLX and the Sarcoplasmic Reticulum Ca2+ Pump SERCA in Cardiomyocytes
Source: Int J Mol Sci. 2022 Jul 19;23(14):7948. doi: 10.3390/ijms23147948 (PMC9317594; doi:10.3390/ijms23147948)
Supplement: Supplementary file 1 [file ijms-23-07948-s001.zip › ijms-1802194-supplementary.pdf]

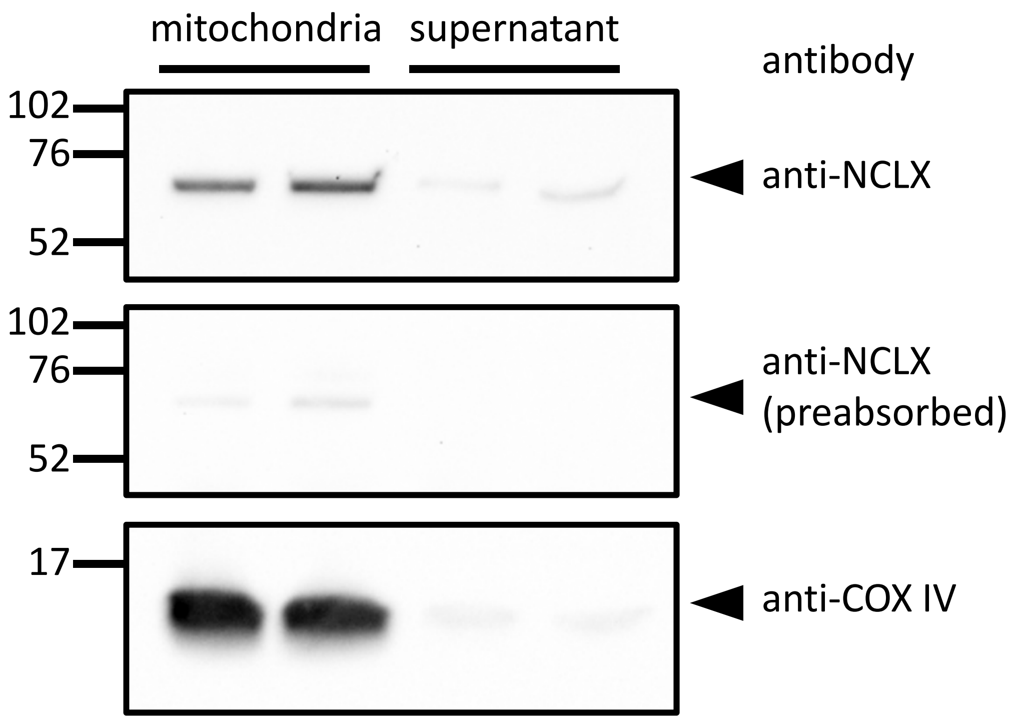


**Figure S1.** Western blot analyses of mouse ventricular mitochondria. Mitochondria were isolated from mouse ventricle using Mitochondria Isolation Kit for Tissue (abcam) according to the manufacture's protocol. The isolated mitochondria (25 μg) and the supernatant fraction (25 μg) from two different mice were used for the experiments. Identity of mitochondria was confirmed by using anti-COX IV antibody. Pre-incubation of the anti-NCLX antibody with excess amount of antigen peptide almost diminished the signal, shown as "preabsorbed".

A

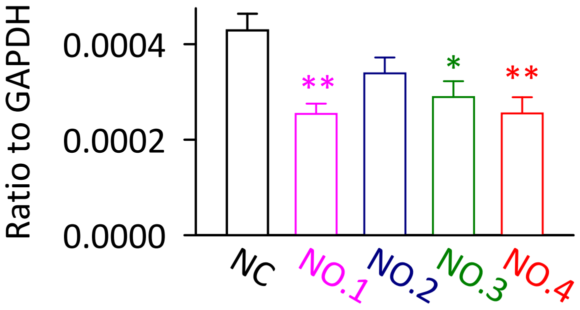


| | | |
|---|---|---|
| NCLX | forward | 5'-TGGCGCTCCAGACATCTTCA-3' |
| | reverse | 5'-CACCCCTGCACCAAACAGAG-3' |
| GAPDH | forward | 5'-TGTGTCCGTCGTGGATCTGA-3' |
| | reverse | 5'-CCTGCTTCACCACCTTCTTGA-3' |

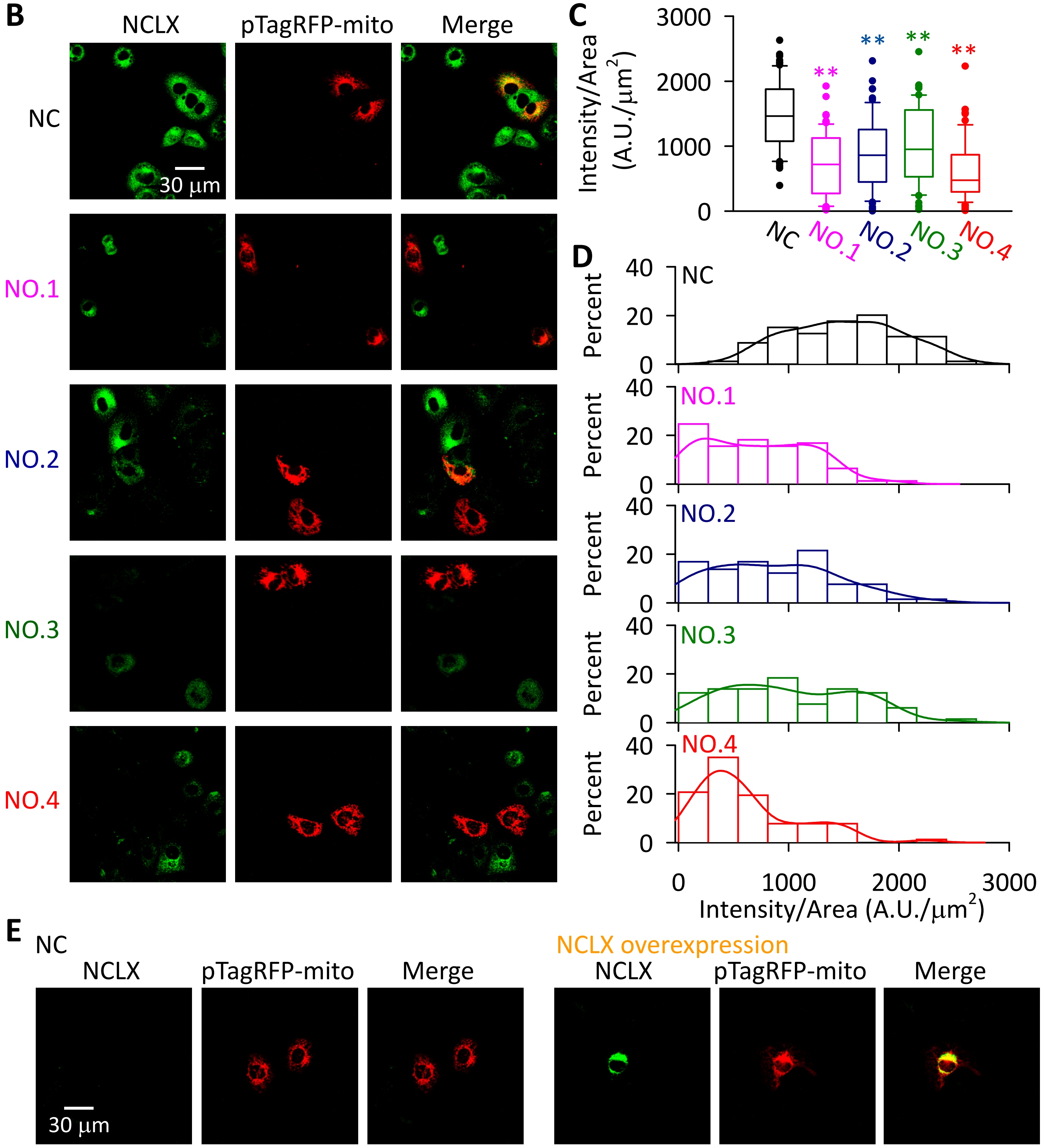

**Figure S2.** Effects of NCLX knockdown in HL-1 cardiomyocytes. **A.** Expression levels of NCLX mRNA in cells transfected with negative control siRNA (NC) or various kinds of NCLX siRNA (NO.1-NO.3, Integrated DNA Technologies; NO.4, Sigma-Aldrich) at a concentration of 50 nM using Lipofectamine RNAiMAX (Thermo Fisher Scientific). Three days after the transfection, total RNA was isolated (RNeasy Plus Micro Kit, QIAGEN) from four different batches and was reverse transcribed (PrimeScript II 1st strand cDNA Synthesis Kit, TAKARA). Real-time PCR was performed with the SYBR green dye technique (Light Cycler 480, Roche Diagnostics) using primers listed on the right. Expression levels of NCLX mRNA normalized with those of GAPDH are expressed as mean $\pm$ s.e.m. of four batches. *$p<0.05$, **$p<0.01$ vs NC. **B-D.** Immunofluorescence analyses of NCLX in cells co-transfected with 0.3 $\mu$g/ml pTagRFP-mito (Evrogen) and 50 nM negative control siRNA (NC) or various kinds of NCLX siRNA (NO.1-NO.4). Three days after the transfection, cells were fixed with 3.6 % paraformaldehyde, were blocked with Image-iT FX Signal Enhancer (Cell Signaling Technology), and were incubated with our custom-made anti-NCLX antibody (1:100). Anti-rabbit IgG Alexa Fluor 488 (1:500, Thermo Fisher Scientific) was used as a secondary antibody. Images were acquired using a laser scanning confocal microscopy (Olympus FV1200) with a x40 objective lens (NA 0.95). Excitation/emission wavelength were 473 (laser 670 V)/485-545 nm and 559 (laser 750 V)/575-675 nm for Alexa Fluor 488 and pTagRFP-mito, respectively. Representative images are shown in **B**. For the analyses, the pTagRFP-mito-positive cells were chosen and the fluorescence intensity of Alexa Fluor 488 was normalized with the cell area using Image J (NIH). Cells incubated with secondary antibody but not with primary antibody were used for calculating background fluorescence intensity/area, which was 0.016 $\pm$ 0.0040 (A.U./$\mu m^2$; n=68 cells). Background fluorescence intensity-subtracted data are summarized in **C**. Data are expressed as mean $\pm$ s.e.m. of 65-79 cells. **$p<0.01$ vs NC. The histograms plus Kernel density (SigmaPlot 14.0) are shown in **D**. **E.** Immunofluorescence analyses of NCLX in cells transfected with 0.3 $\mu$g/ml pTagRFP-mito (Evrogen) and 0.3 $\mu$g/ml mouse NCLX-pCMV6 (Takeuchi and Matsuoka, *Cell Calcium*, 2021). Images for Alexa Fluor 488  were acquired with a lower laser intensity, 550 V compared with 670 V. As a reference, images from negative control siRNA-transfected cells (NC) obtained under the same laser conditions are shown, in which Alexa Fluor 488-positive signals in **B** were completely diminished.

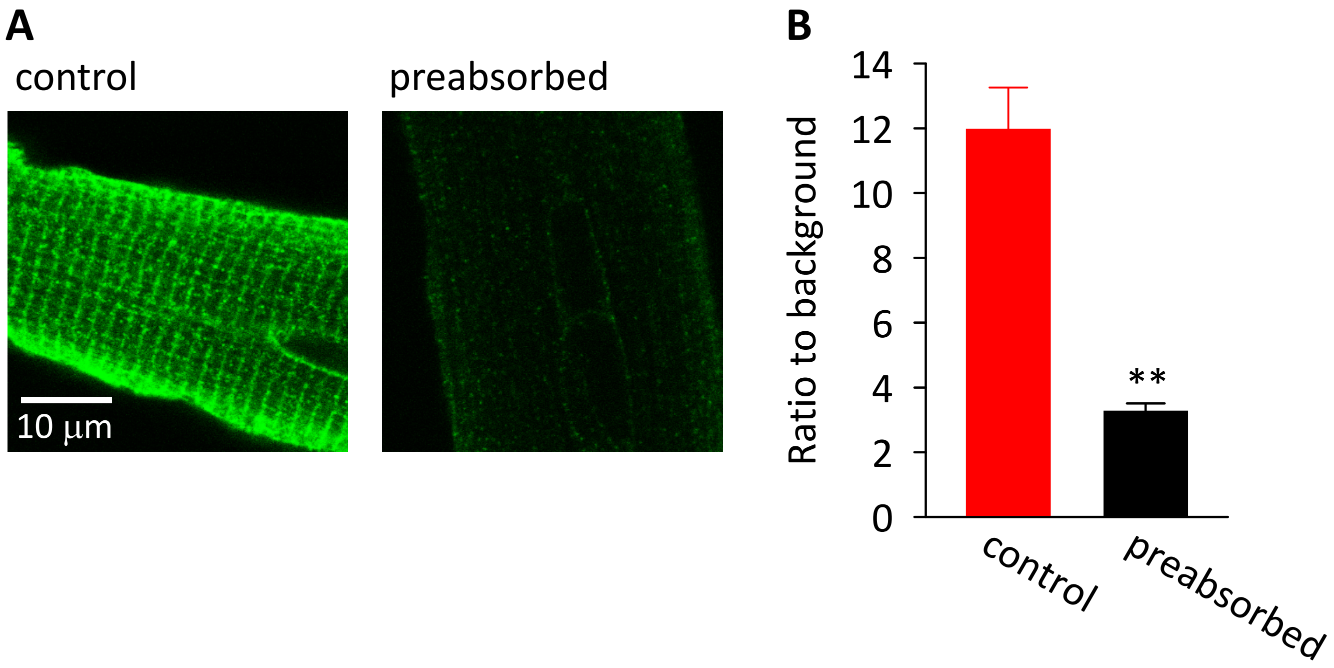


**Figure S3.** Immunofluorescence of NCLX in mouse ventricular myocytes.
**A.** Representative images. Pre-incubation of the anti-NCLX antibody with excess amount of antigen peptide diminished the signal (preabsorbed).
**B.** Fluorescent intensity/area of the cells, which were normalized with those not treated with primary antibody. Data are expressed as mean ± s.e.m. of 5 cells. **$p<0.01$ vs control.

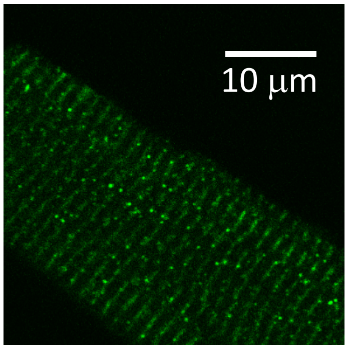


**Figure S4.** Immunofluorescence of MCU in mouse ventricular myocyte.

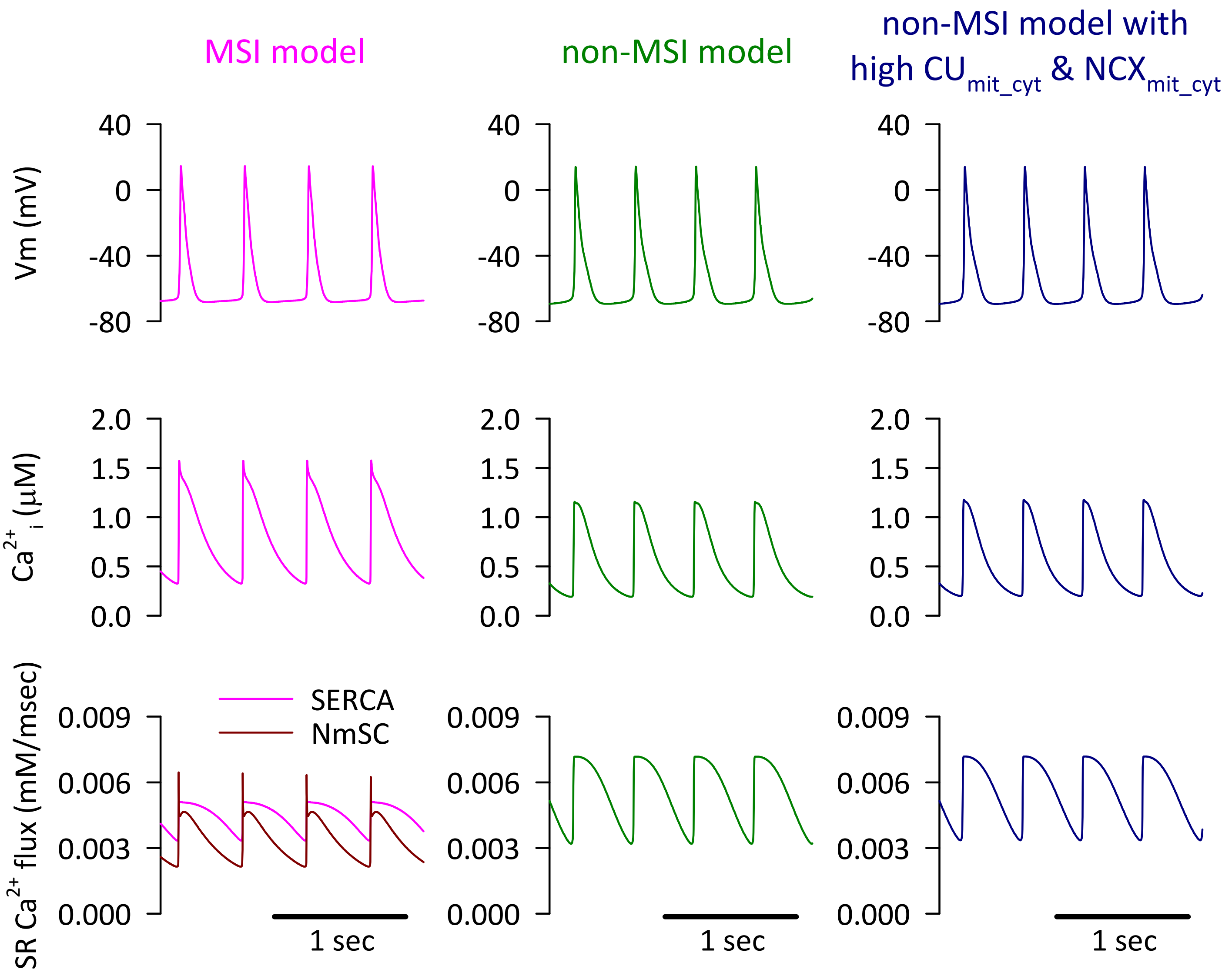


**Figure S5.** Configurations of action potentials, cytosolic $Ca^{2+}$ ($Ca^{2+}{}_i$) transients, and SR $Ca^{2+}$ fluxes via SERCA and NmSC in MSI model (magenta), non-MSI model (green) and non-MSI model with high $CU_{mit\_cyt}$ & $NCX_{mit\_cyt}$ (dark blue).

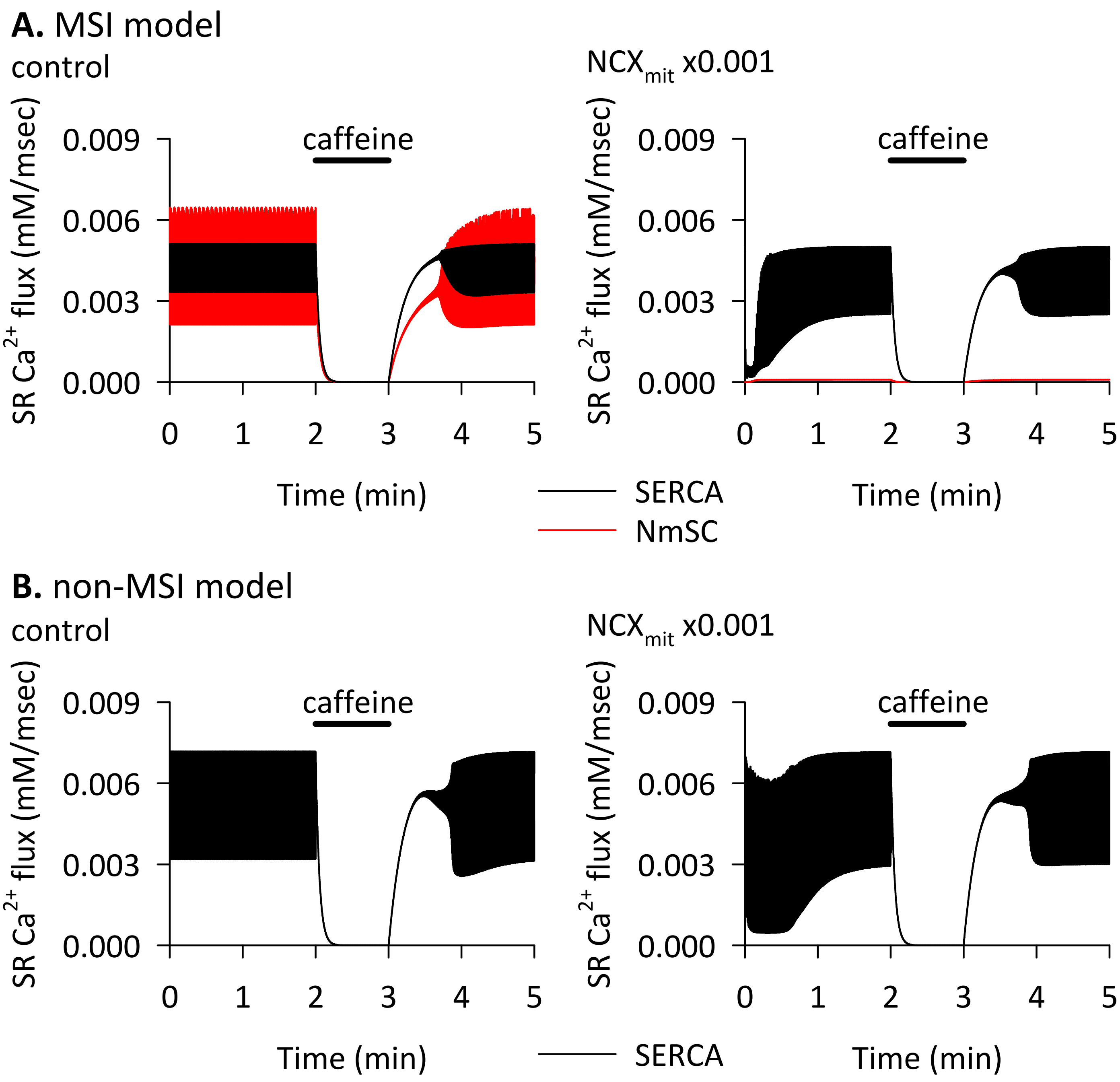


**Figure S6.** Simulation of the effects of $NCX_{mit}$ scaling factor reduction to 0.001 on the time courses of SR $Ca^{2+}$ fluxes via SERCA (black lines) and NmSC (red lines) during the caffeine application protocol using MSI model (**A**) and non-MSI model (**B**).

# Supplementary Material

## Description of the Mathematical Model of HL-1 Cardiomyocytes with/without Mitochondria–SR Interaction (MSI Model/Non-MSI Model or Non-MSI model with high $\mathbf{CU_{mit\_cyt}}$ & $\mathbf{NCX_{mit\_cyt}}$)

This is a supplemental material that describes details of the mathematical model of HL-1 cardiomyocytes with/without mitochondria–sarcoplasmic reticulum (SR) interaction (MSI model/non-MSI model or non-MSI model with high $CU_{mit\_cyt}$ & $NCX_{mit\_cyt}$).

Based on the biased distributions of mitochondrial and SR $Ca^{2+}$ handling proteins (Figure 6E in the main text and De La Fuente et al., *J Biol Chem*, 2016), the MSI model was updated from the previous HL-1 cell model (Takeuchi et al., *Sci Rep*, 2013), described as follows. 1) The NCLX–SERCA complex (NmSC) and $CU_{mit}$ facing junctional space ($CU_{mit\_JS}$) were newly implemented. 2) The scaling factor of NmSC was set as 7/3 of that of $NCX_{mit}$ facing cytoplasm ($NCX_{mit\_cyt}$), according to the Manders' coefficient of NCLX overlapping with SERCA2, ~0.7 (Figure 6E in the main text). 2) The scaling factor of $CU_{mit\_JS}$ was set to the same value as that of $CU_{mit}$ facing cytoplasm ($CU_{mit\_cyt}$), according to the super-resolution images of MCU, in which about half of MCU was localized near RyR2 at junctional SR (De La Fuente et al., *J Biol Chem*, 2016). 3) Functional coupling of NCLX and SERCA via NmSC was expressed by assuming that $Ca^{2+}$, that is extruded through NmSC from mitochondria, directly enters SR. 4) The 30% of SERCA was replaced with NmSC, according to the Manders' coefficient of SERCA overlapping with NCLX, ~0.3 (Figure 6E in the main text). Several parameters for mitochondrial and SR $Ca^{2+}$ fluxes were modified for the firing rate and cytosolic $Ca^{2+}$ dynamics to become within the range of experimental data.

The original HL-1 cell model defined the mitochondrial membrane potential ($\Delta\Psi$) as a constant value (−150 mV) regardless of the mitochondrial $Ca^{2+}$ level. This made the $CU_{mit}$ to be continuously active even under the mitochondrial $Ca^{2+}$-overloaded condition, although mitochondrial $Ca^{2+}$ overload is known to depolarize $\Delta\Psi$ to impede $CU_{mit}$. As a result, unrealistically high level of mitochondrial $Ca^{2+}$ occurred especially when the $NCX_{mit}$—the only pathway for extruding mitochondrial $Ca^{2+}$ in the model—was inhibited in the long time (5 min) simulation of caffeine application. In order to prevent this, we newly implemented the equations for mitochondrial $Ca^{2+}$ overload-mediated $CU_{mit}$ inhibition (see $I_{CUmit}$ in Table S5).

The scheme of the MSI model is shown in the Figure 8 in the main text. The non-MSI model was constructed by removing NmSC and $CU_{mit\_JS}$. The non-MSI model with increased $CU_{mit\_cyt}$ & $NCX_{mit\_cyt}$ was constructed by increasing the scaling factor of $CU_{mit\_cyt}$ and $NCX_{mit\_cyt}$ by 2 and 10/3 times, respectively, in the same manner as the MSI model. This model was created to confirm that the biased distributions of $CU_{mit}$ and $NCX_{mit}$, not their scaling factors—i.e. expression levels—are important for reproducing the experimental data on NCLX inhibition.

The models were created with Visual C# (Microsoft Visual Studio 2019) and the ordinary differential equations were integrated by Runge-Kutta method with adaptive time step. Units are mM for concentration and msec for time. Mathematical formulas are described in the following tables (Tables S1-S8). The caffeine application was simulated by increasing the open probability of RyR—multiplying the "koCa" in RyR (see $I_{RyR}$ in Table S4) by 11—and by blocking SERCA with time constant of 3000 msec. The caffeine effect was reversed with time constant of 15000 msec. In the MSI model, $Ca^{2+}$ extruded via NmSC during caffeine application was assumed to leak to cytoplasm.

## Table S1. Abbreviations

| | |
|---|---|
| R | Gas constant, 8.314472 C mV/K/mmol |
| F | Faraday's constant, 96.48653415 C/mmol |
| T | Absolute temperature, 310 K |
| $Ca_o$ | Extracellular $Ca^{2+}$, 1.8 mM |
| $Ca_i$ | Cytoplasmic $Ca^{2+}$ concentration in mM |
| $Ca_{JS}$ | Junctional space $Ca^{2+}$ concentration in mM |
| $Ca_{SL}$ | Subsarcolemmal space $Ca^{2+}$ concentration in mM |
| $Ca_{SR}$ | Sarcoplasmic reticulum $Ca^{2+}$ concentration in mM |
| $Ca_{mit}$ | Mitochondrial free $Ca^{2+}$ concentration in mM |
| $Ca_{totalmit}$ | Mitochondrial total $Ca^{2+}$ concentration in mM |
| $Na_o$ | Extracellular $Na^+$ concentration, 140 mM |
| $Na_i$ | Cytoplasmic $Na^+$ concentration, 15.0 mM |
| $Na_{mit}$ | Mitochondrial $Na^+$ concentration, 4.0 mM |
| $K_o$ | Extracellular $K^+$ concentration, 5.4 mM |
| $K_i$ | Cytoplasmic $K^+$ concentration, 120 mM |
| $Cl_o$ | Extracellular $Cl^-$ concentration, 150 mM |
| $Cl_i$ | Cytoplasmic $Cl^-$ concentration, 15 mM |
| $\Delta\Psi$ | Mitochondrial membrane potential, −150 mV |
| $Mg_i$ | Cytoplasmic $Mg^{2+}$ concentration, 1.0 mM |

## Table S2. Cell property

| | Abbreviation | Value | Unit |
|---|---|---|---|
| Total cellular volume | $Vol_i$ | 33000 | $\mu m^3$ |
| Cytoplasm volume | $Vol_{cyt}$ | $0.65 \cdot Vol_i$ | $\mu m^3$ |
| Junctional space volume | $Vol_{JS}$ | $4.0 \cdot 10^{-4} \cdot Vol_i$ | $\mu m^3$ |
| Subsarcolemmal space volume | $Vol_{SL}$ | $0.013 \cdot Vol_i$ | $\mu m^3$ |
| SR volume | $Vol_{SR}$ | $0.023 \cdot Vol_i$ | $\mu m^3$ |
| Mitochondrial volume | $Vol_{mit}$ | $0.2 \cdot Vol_i$ | $\mu m^3$ |

| | | | |
|---|---|---|---|
| Cell capacitance | Cm | 40 | pF |
| Fractional current of junctional space | $F_{JS}$ | 0.11 | |
| Fractional current of subsarcolemmal space | $F_{SL}$ | $1-F_{JS}$ | |
| Fractional current through $I_{CaL}$ of junctional space | $F_{JSCa}$ | 0.9 | |
| Fractional current through $I_{CaL}$ of subsarcolemmal space | $F_{SLCa}$ | $1-F_{JSCa}$ | |

# Table S3. Ion fluxes at junctional and subsarcolmmal membrane

$I_{Na}$; Voltage-dependent $Na^+$ current

| | Abbreviation | Value | Unit |
|---|---|---|---|
| conductance | $G_{Na}$ | 23 | nS/pF |

$$m_{ss} = \frac{1}{\left(1 + \exp\left(-\frac{56.86 + Vm}{9.03}\right)\right)^2}$$

$$\tau_m = 0.1292 \cdot \exp\left(-\left(\frac{Vm + 45.79}{15.54}\right)^2\right) + 0.06487 \cdot \exp\left(-\left(\frac{Vm - 4.823}{51.12}\right)^2\right)$$

$$h_{ss} = \frac{1}{\left(1 + \exp\left(\frac{Vm + 71.55}{7.43}\right)\right)^2}$$

$$\alpha_h = \begin{cases} 0.057 \cdot \exp\left(-\frac{Vm + 80}{6.8}\right), & Vm < -40\, mV \\ 0, & otherwise \end{cases}$$

$$\beta_h = \begin{cases} 2.7 \cdot \exp(0.079 \cdot Vm) + 3.1 \cdot 10^5 \cdot \exp(0.3485 \cdot Vm), & Vm < -40\, mV \\ \frac{0.77}{0.13 \cdot \left(1 + exp\left(-\frac{Vm + 10.66}{11.1}\right)\right)}, & otherwise \end{cases}$$

$$\tau_h = \frac{1}{\alpha_h + \beta_h}$$

$$j_{ss} = \frac{1}{\left(1 + \exp\left(\frac{Vm + 71.55}{7.43}\right)\right)^2}$$

$$\alpha_j = \begin{cases} \frac{\left(-2.5428 \cdot 10^4 \cdot \exp(0.2444 \cdot Vm) - 6.948 \cdot 10^{-6} \cdot \exp(-0.04391 \cdot Vm)\right) \cdot (Vm + 37.78)}{1 + \exp\left(0.311 \cdot (Vm + 79.23)\right)}, & Vm < -40\, mV \\ 0, & otherwise \end{cases}$$

$$\beta_j = \begin{cases} \frac{0.02424 \cdot \exp(-0.01052 \cdot Vm)}{1 + \exp\left(-0.1378 \cdot (Vm + 40.14)\right)}, & Vm < -40\, mV \\ \frac{0.6 \cdot \exp(0.057 \cdot Vm)}{1 + \exp\left(-0.1 \cdot (Vm + 32)\right)}, & otherwise \end{cases}$$

$$\tau_j = \frac{1}{\alpha_j + \beta_j}$$

$$\frac{dm}{dt} = \frac{m_{ss} - m}{\tau_m}$$

$$\frac{dh}{dt} = \frac{h_{ss} - h}{\tau_h}$$

$$\frac{dj}{dt} = \frac{j_{ss} - j}{\tau_j}$$

$$INa_{JS} = F_{JS} \cdot G_{Na} \cdot m^3 \cdot h \cdot j \cdot (Vm - E_{Na})$$

$$INa_{SL} = F_{SL} \cdot G_{Na} \cdot m^3 \cdot h \cdot j \cdot (Vm - E_{Na})$$

$$I_{Na} = INa_{JS} + INa_{SL}$$

$I_{NaK}$; Sarcolemmal $Na^+/K^+$ pump current

| | Abbreviation | Value | Unit |
|---|---|---|---|
| amplitude | $A_{NaK}$ | 1.26 | pA/pF |
| dissociation constant | $Km_{Nai}$ | 11.0 | mM |
| dissociation constant | $Km_{Ko}$ | 1.5 | mM |

$$\sigma = \frac{\exp\left(\frac{Na_o}{67.3}\right) - 1}{7}$$

$$f_{NaK} = \frac{1}{1 + 0.1245 \cdot \exp\left(-0.1 \cdot Vm \cdot \frac{F}{RT}\right) + 0.0365 \cdot \sigma \cdot \exp\left(-Vm \cdot \frac{F}{RT}\right)}$$

$$INaK_{JS} = F_{JS} \cdot \frac{A_{NaK} \cdot f_{NaK}}{1 + \left(\frac{Km_{Na_i}}{Na_i}\right)^4} \cdot \frac{K_o}{K_o + Km_{K_o}}$$

$$INaK_{SL} = F_{SL} \cdot \frac{A_{NaK} \cdot f_{NaK}}{1 + \left(\frac{Km_{Na_i}}{Na_i}\right)^4} \cdot \frac{K_o}{K_o + Km_{K_o}}$$

$$I_{NaK} = INaK_{JS} + INaK_{SL}$$

$I_{Kr}$; Rapidly activating $K^+$ current

| | Abbreviation | Value | Unit |
|---|---|---|---|
| conductance | $G_{Kr}$ | 0.73 | nS/pF |

$$xkr_{ss} = \frac{1}{1 + \exp\left(-\frac{Vm + 15}{6}\right)}$$

$$\tau_{xkr} = 2.5 \cdot \left(31.18 + 217.18 \cdot \exp\left(-\left(\frac{Vm + 20.1376}{22.1996}\right)^2\right)\right)$$

$$rkr = \frac{1}{1 + \exp\left(\frac{Vm + 55}{24}\right)}$$

$$\frac{dxkr}{dt} = \frac{xkr_{ss} - xkr}{\tau_{xkr}}$$

$$IKr_{JS} = F_{JS} \cdot G_{Kr} \cdot \sqrt{\frac{K_o}{5.4}} \cdot xkr \cdot rkr \cdot (Vm - E_K)$$

$$IKr_{SL} = F_{SL} \cdot G_{Kr} \cdot \sqrt{\frac{K_o}{5.4}} \cdot xkr \cdot rkr \cdot (Vm - E_K)$$

$$I_{Kr} = IKr_{JS} + IKr_{SL}$$

$I_{Ks}$; Slowly activating $K^+$ current

| | Abbreviation | Value | Unit |
|---|---|---|---|
| conductance | $G_{Ks}$ | 0.0035 | nS/pF |
| | pNaK | 0.01833 | |

$$xks_{ss} = \frac{1}{1 + \exp\left(-\frac{Vm + 3.8}{14.25}\right)}$$

$$\tau_{xks} = \frac{990.1}{1 + \exp\left(-\frac{Vm + 2.436}{14.12}\right)}$$

$$\frac{dxks}{dt} = \frac{xks_{ss} - xks}{\tau_{xks}}$$

$$E_{Ks} = \frac{RT}{F} \ln\left(\frac{K_o + pNaK \cdot Na_o}{K_i + pNaK \cdot Na_i}\right)$$

$$IKs_{JS} = F_{JS} \cdot G_{Ks} \cdot xks^2 \cdot (Vm - E_{Ks})$$

$$IKs_{SL} = F_{SL} \cdot G_{Ks} \cdot xks^2 \cdot (Vm - E_{Ks})$$

$$I_{Ks} = IKs_{JS} + IKs_{SL}$$

$I_{Kp}$; Plateau $K^+$ current

| | Abbreviation | Value | Unit |
|---|---|---|---|
| conductance | $G_{Kp}$ | 0.002 | nS/pF |

$$kp_{kp} = \frac{1}{1 + \exp\left(7.488 - \frac{Vm}{5.98}\right)}$$

$$IKp_{JS} = F_{JS} \cdot G_{Kp} \cdot kp_{kp} \cdot (Vm - E_K)$$

$$IKp_{SL} = F_{SL} \cdot G_{Kp} \cdot kp_{kp} \cdot (Vm - E_K)$$

| |
|---|
| $I_{Kp} = IKp_{JS} + IKp_{SL}$ |

$I_{to}$; Transient outward $K^+$ current

| | Abbreviation | Value | Unit |
|---|---|---|---|
| conductance | $G_{to}$ | 0.12375 | nS/pF |

$$xto_{ss} = \frac{1}{1 + \exp\left(-\frac{Vm - 1}{11}\right)}$$

$$\tau_{xto} = 1.5 + 3.5 \cdot \exp\left(-\left(\frac{Vm}{30}\right)^2\right)$$

$$yto_{ss} = \frac{1}{1 + \exp\left(\frac{Vm + 40.5}{11.5}\right)}$$

$$\tau_{yto} = 21.21 + 38.4525 \cdot \exp\left(-\left(\frac{Vm + 52.45}{15.8827}\right)^2\right)$$

$$\frac{dxto}{dt} = \frac{xto_{ss} - xto}{\tau_{xto}}$$

$$\frac{dyto}{dt} = \frac{yto_{ss} - yto}{\tau_{yto}}$$

$$Ito_{JS} = F_{JS} \cdot G_{to} \cdot xto \cdot yto \cdot (Vm - E_K)$$

$$Ito_{SL} = F_{SL} \cdot G_{to} \cdot xto \cdot yto \cdot (Vm - E_K)$$

$$I_{to} = Ito_{JS} + Ito_{SL}$$

$I_{Kur}$; Ultrarapid delayed rectifier $K^+$ current

| | Abbreviation | Value | Unit |
|---|---|---|---|
| conductance | $G_{Kur}$ | 0.035 | nS/pF |

$$xKur_{ss} = \frac{1}{1 + \exp\left(-\frac{Vm + 6}{8.6}\right)}$$

$$\tau_{xKur} = 0.5 + \frac{9}{1 + \exp\left(\frac{Vm + 5}{12}\right)}$$

$$yKur_{ss} = \frac{1}{1 + \exp\left(\frac{Vm + 7.5}{10}\right)}$$

$$\tau_{yKur} = 3050 + \frac{590}{1 + \exp\left(\frac{Vm + 60}{10}\right)}$$

$$\frac{dxKur}{dt} = \frac{xKur_{ss} - xKur}{\tau_{xKur}}$$

$$\frac{dyKur}{dt} = \frac{yKur_{ss} - yKur}{\tau_{yKur}}$$

| |
|---|
| $IKur_{JS} = F_{JS} \cdot G_{Kur} \cdot xKur \cdot yKur \cdot (Vm - E_K)$<br>$IKur_{SL} = F_{SL} \cdot G_{Kur} \cdot xKur \cdot yKur \cdot (Vm - E_K)$<br>$I_{Kur} = IKur_{JS} + IKur_{SL}$ |

$I_{K1}$; Inward rectifier $K^+$ current

| | Abbreviation | Value | Unit |
|---|---|---|---|
| conductance | $G_{K1}$ | 0.15 | nS/pF |

$$\alpha_{K1} = \frac{1.02}{1 + \exp\left(0.2385 \cdot (Vm - E_K - 59.215)\right)}$$

$$\beta_{K1} = \frac{0.49124 \cdot \exp\left(0.08032 \cdot (Vm + 5.476 - E_K)\right) + \exp\left(0.06175 \cdot (Vm - 594.31 - E_K)\right)}{1 + \exp\left(-0.5143 \cdot (Vm + 4.753 - E_K)\right)}$$

$$K1_{ss} = \frac{\alpha_{K1}}{\alpha_{K1} + \beta_{K1}}$$

$$IK1_{JS} = F_{JS} \cdot G_{K1} \cdot \sqrt{\frac{K_o}{5.4}} \cdot K1_{ss}(Vm - E_K)$$

$$IK1_{SL} = F_{SL} \cdot G_{K1} \cdot \sqrt{\frac{K_o}{5.4}} \cdot K1_{ss}(Vm - E_K)$$

$$I_{K1} = IK1_{JS} + IK1_{SL}$$

$I_{ClCa}$; $Ca^{2+}$ activated $Cl^-$ current

| | Abbreviation | Value | Unit |
|---|---|---|---|
| conductance | $G_{ClCa}$ | 0.0548125 | nS/pF |
| | $Kd_{ClCa}$ | 0.1 | |

$$IClCa_{JS} = \frac{F_{JS} \cdot G_{ClCa} \cdot (Vm - E_{Cl})}{1 + \frac{Kd_{ClCa}}{Ca_{JS}}}$$

$$IClCa_{SL} = \frac{F_{SL} \cdot G_{ClCa} \cdot (Vm - E_{Cl})}{1 + \frac{Kd_{ClCa}}{Ca_{SL}}}$$

$$I_{ClCa} = IClCa_{JS} + IClCa_{SL}$$

$I_{Clb}$; Background $Cl^-$ current

| | Abbreviation | Value | Unit |
|---|---|---|---|
| conductance | $G_{Clb}$ | 0.0045 | nS/pF |

$$ICl b_{JS} = F_{JS} \cdot G_{Clb} \cdot (Vm - E_{Cl})$$
$$ICl b_{SL} = F_{SL} \cdot G_{Clb} \cdot (Vm - E_{Cl})$$
$$I_{Clb} = ICl b_{JS} + ICl b_{SL}$$

$I_{CaL}$; L-type $Ca^{2+}$ current

| | Abbreviation | Value | Unit |
|---|---|---|---|
| permeability | $P_{Ca}$ | 0.200475 | cm/sec |
| permeability | $P_{Na}$ | $5.56875 \cdot 10^{-6}$ | cm/sec |
| permeability | $P_K$ | $1.002375 \cdot 10^{-4}$ | cm/sec |

$$d_{ss} = \frac{1}{1 + \exp\left(-\frac{Vm + 1}{6.0}\right)}$$
$$\tau_d = d_{ss} \cdot \frac{1 - \exp\left(-\frac{Vm + 5}{6.0}\right)}{0.035 \cdot (Vm + 5)}$$
$$f_{ss} = \frac{1}{1 + \exp\left(\frac{Vm + 30}{9}\right)} + \frac{0.6}{1 + \exp\left(\frac{50 - Vm}{20}\right)}$$
$$\tau_f = \frac{2}{0.0197 \cdot \exp\left(-\left(0.0337 \cdot (Vm + 14.5)\right)^2\right) + 0.02}$$
$$\frac{dd}{dt} = \frac{d_{ss} - d}{\tau_d}$$
$$\frac{df}{dt} = \frac{f_{ss} - f}{\tau_f}$$
$$\frac{dfCaB_{JS}}{dt} = 1.7 \cdot Ca_{JS} \cdot \left(1 - fCaB_{JS}\right) - 11.9 \cdot 10^{-3} \cdot fCaB_{JS}$$
$$\frac{dfCaB_{SL}}{dt} = 1.7 \cdot Ca_{SL} \cdot (1 - fCaB_{SL}) - 11.9 \cdot 10^{-3} \cdot fCaB_{SL}$$
$$ICaL_{CaJS} = F_{JSCa} \cdot d \cdot f \cdot \left(1 - fCaB_{JS}\right) \cdot \frac{PCa \cdot 4 \cdot Vm \cdot F \cdot \frac{F}{RT} \cdot \left(0.341 \cdot Ca_{JS} \cdot \exp\left(2 \cdot Vm \cdot \frac{F}{RT}\right) - 0.341 \cdot Ca_o\right)}{\exp\left(2 \cdot Vm \cdot \frac{F}{RT}\right) - 1}$$
$$ICaL_{CaSL} = F_{SLCa} \cdot d \cdot f \cdot (1 - fCaB_{SL}) \cdot \frac{PCa \cdot 4 \cdot Vm \cdot F \cdot \frac{F}{RT} \cdot \left(0.341 \cdot Ca_{SL} \cdot \exp\left(2 \cdot Vm \cdot \frac{F}{RT}\right) - 0.341 \cdot Ca_o\right)}{\exp\left(2 \cdot Vm \cdot \frac{F}{RT}\right) - 1}$$
$$ICaL_{Ca} = ICaL_{CaJS} + ICaL_{CaSL}$$
$$ICaL_{NaJS} = F_{JSCa} \cdot d \cdot f \cdot \left(1 - fCaB_{JS}\right) \cdot \frac{PNa \cdot Vm \cdot F \cdot \frac{F}{RT} \cdot \left(0.75 \cdot Na_i \cdot \exp\left(Vm \cdot \frac{F}{RT}\right) - 0.75 \cdot Na_o\right)}{\exp\left(Vm \cdot \frac{F}{RT}\right) - 1}$$

$$ICaL_{NaSL} = F_{SLCa} \cdot d \cdot f \cdot (1 - fCaB_{SL}) \cdot \frac{PNa \cdot Vm \cdot F \cdot \frac{F}{RT} \cdot \left(0.75 \cdot Na_i \cdot \exp\left(Vm \cdot \frac{F}{RT}\right) - 0.75 \cdot Na_o\right)}{\exp\left(Vm \cdot \frac{F}{RT}\right) - 1}$$

$$ICaL_{Na} = ICaL_{NaJS} + ICaL_{NaSL}$$

$$ICaL_{KJS} = F_{JSCa} \cdot d \cdot f \cdot \left(1 - fCaB_{JS}\right) \cdot \frac{PK \cdot Vm \cdot F \cdot \frac{F}{RT} \cdot \left(0.75 \cdot K_i \cdot \exp\left(Vm \cdot \frac{F}{RT}\right) - 0.75 \cdot K_o\right)}{\exp\left(Vm \cdot \frac{F}{RT}\right) - 1}$$

$$ICaL_{KSL} = F_{SLCa} \cdot d \cdot f \cdot (1 - fCaB_{SL}) \cdot \frac{PK \cdot Vm \cdot F \cdot \frac{F}{RT} \cdot \left(0.75 \cdot K_i \cdot \exp\left(Vm \cdot \frac{F}{RT}\right) - 0.75 \cdot K_o\right)}{\exp\left(Vm \cdot \frac{F}{RT}\right) - 1}$$

$$ICaL_K = ICaL_{KJS} + ICaL_{KSL}$$

$$I_{CaL} = ICaL_{Ca} + ICaL_{Na} + ICaL_K$$

$I_{CaT}$; T-type $Ca^{2+}$ current

| | Abbreviation | Value | Unit |
|---|---|---|---|
| conductance | $G_{CaT}$ | 0.4122 | nS/pF |

$$d_{ss} = \frac{1}{1 + \exp\left(-\frac{Vm + 30}{6.0}\right)}$$

$$\tau_d = \frac{1}{1.068 \cdot \exp\left(\frac{Vm + 26.3}{30}\right) + 1.068 \cdot \exp\left(-\frac{Vm + 26.3}{30}\right)}$$

$$f_{ss} = \frac{1}{1 + \exp\left(\frac{Vm + 48}{7}\right)}$$

$$\tau_f = \frac{1}{0.0153 \cdot \exp\left(-\frac{Vm + 61.7}{83.3}\right) + 0.015 \cdot \exp\left(\frac{Vm + 61.7}{30}\right)}$$

$$\frac{dd}{dt} = \frac{d_{ss} - d}{\tau_d}$$

$$\frac{df}{dt} = \frac{f_{ss} - f}{\tau_f}$$

$$ICaT_{JS} = F_{JS} \cdot G_{CaT} \cdot d \cdot f \cdot (Vm - 45)$$

$$ICaT_{SL} = F_{SL} \cdot G_{CaT} \cdot d \cdot f \cdot (Vm - 45)$$

$$I_{CaT} = ICaT_{JS} + ICaT_{SL}$$

$I_{pCa}$; Sarcolemmal $Ca^{2+}$ pump current

| | Abbreviation | Value | Unit |
|---|---|---|---|
| amplitude | $A_{pCa}$ | 0.0673 | pA/pF |
| dissociation constant | $Km_{pCa}$ | $5.0 \cdot 10^{-4}$ | mM |

$$IpCa_{JS} = \frac{F_{JS} \cdot A_{pCa} \cdot Ca_{JS}^{1.6}}{Km_{pCa}^{1.6} + Ca_{JS}^{1.6}}$$

$$IpCa_{SL} = \frac{F_{SL} \cdot A_{pCa} \cdot Ca_{SL}^{1.6}}{Km_{pCa}^{1.6} + Ca_{SL}^{1.6}}$$

$$I_{pCa} = IpCa_{JS} + IpCa_{SL}$$

$I_{Cab}$; Background $Ca^{2+}$ current

| | Abbreviation | Value | Unit |
|---|---|---|---|
| conductance | $G_{Cab}$ | $5.513 \cdot 10^{-4}$ | nS/pF |

$$ICab_{JS} = F_{JS} \cdot G_{Cab} \cdot (Vm - E_{CaJS})$$

$$ICab_{SL} = F_{SL} \cdot G_{Cab} \cdot (Vm - E_{CaSL})$$

$$I_{Cab} = ICab_{JS} + ICab_{SL}$$

$I_{NCX}$; Sarcolemmal $Na^{+}$-$Ca^{2+}$ exchange current

| | Abbreviation | Value | Unit |
|---|---|---|---|
| amplitude | $A_{NCX}$ | 4.0 | pA/pF |
| dissociation constant | $Kd_{act}$ | $2.25 \cdot 10^{-4}$ | mM |
| dissociation constant | $Km_{Cai}$ | 0.00359 | mM |
| dissociation constant | $Km_{Cao}$ | 1.3 | mM |
| dissociation constant | $Km_{Nai}$ | 12.29 | mM |
| dissociation constant | $Km_{Nao}$ | 87.5 | mM |
| | nu | 0.27 | |
| | $k_{sat}$ | 0.32 | |

$$Ka_{JS} = \frac{1}{1 + \left(\frac{Kd_{act}}{Ca_{JS}}\right)^2}$$

$$Ka_{SL} = \frac{1}{1 + \left(\frac{Kd_{act}}{Ca_{SL}}\right)^2}$$

$$s1 = \exp\left(nu \cdot Vm \cdot \frac{F}{RT}\right) \cdot Na_i^3 \cdot Ca_o$$

$$s2_{JS} = \exp\left((nu - 1) \cdot Vm \cdot \frac{F}{RT}\right) \cdot Na_o^3 \cdot Ca_{JS}$$

$$s2_{SL} = \exp\left((nu - 1) \cdot Vm \cdot \frac{F}{RT}\right) \cdot Na_o^3 \cdot Ca_{SL}$$

$$s3_{JS} = Km_{Ca_i} \cdot Na_o^3 \cdot \left(1 + \left(\frac{Na_i}{Km_{Na_i}}\right)^3\right) + Km_{Na_o}^3 \cdot Ca_{JS} \cdot \left(1 + \frac{Ca_{JS}}{Km_{Ca_i}}\right) + Km_{Ca_o} \cdot Na_i^3 + Na_i^3 \cdot Ca_o + Na_o^3 \cdot Ca_{JS}$$

$$s3_{SL} = Km_{Ca_i} \cdot Na_o^3 \cdot \left(1 + \left(\frac{Na_i}{Km_{Na_i}}\right)^3\right) + Km_{Na_o}^3 \cdot Ca_{SL} \cdot \left(1 + \frac{Ca_{SL}}{Km_{Ca_i}}\right) + Km_{Ca_o} \cdot Na_i^3 + Na_i^3 \cdot Ca_o + Na_o^3 \cdot Ca_{SL}$$

$$INCX_{JS} = \frac{F_{JS} \cdot A_{NCX} \cdot Ka_{JS} \cdot (s1 - s2_{JS})}{s3_{JS} \cdot \left(1 + k_{sat} \cdot \exp\left((nu-1) \cdot Vm \cdot \frac{F}{RT}\right)\right)}$$

$$INCX_{SL} = \frac{F_{SL} \cdot A_{NCX} \cdot Ka_{SL} \cdot (s1 - s2_{SL})}{s3_{SL} \cdot \left(1 + k_{sat} \cdot \exp\left((nu-1) \cdot Vm \cdot \frac{F}{RT}\right)\right)}$$

$$I_{NCX} = INCX_{JS} + INCX_{SL}$$

$I_{ha}$; Hyperpolarization-activated cation current

| | Abbreviation | Value | Unit |
|---|---|---|---|
| conductance | $G_{ha}$ | 0.2 | nS/pF |

$$y_{ss} = \frac{1}{1 + \exp\left(\frac{Vm + 70}{13.5}\right)}$$

$$\tau_y = \frac{1}{\exp\left(-\frac{Vm + 198}{15}\right) + \exp\left(\frac{Vm - 130}{17}\right)}$$

$$\frac{dy}{dt} = \frac{y_{ss} - y}{\tau_y}$$

$$Iha_{NaJS} = F_{JS} \cdot G_{ha} \cdot 0.3833 \cdot (Vm - E_{Na}) \cdot y^2$$

$$Iha_{KJS} = F_{JS} \cdot G_{ha} \cdot 0.6167 \cdot (Vm - E_K) \cdot y^2$$

$$Iha_{JS} = Iha_{NaJS} + Iha_{KJS}$$

$$Iha_{NaSL} = F_{SL} \cdot G_{ha} \cdot 0.3833 \cdot (Vm - E_{Na}) \cdot y^2$$

$$Iha_{KSL} = F_{SL} \cdot G_{ha} \cdot 0.6167 \cdot (Vm - E_K) \cdot y^2$$

$$Iha_{SL} = Iha_{NaSL} + Iha_{KSL}$$

$$I_{ha} = Iha_{JS} + Iha_{SL}$$

# Buffers;

## Cytosolic $Ca^{2+}$ buffers

| | Abbreviation | Value | Unit |
|---|---|---|---|
| association constant | $kon_{TnClow}$ | 32.7 | /mM· msec |
| dissociation constant | $koff_{TnClow}$ | 0.0196 | / msec |
| total $TnC_{low}$ concentration | $Bmax_{TnClow}$ | 0.07 | mM |
| association constant | $kon_{TnChCa}$ | 2.37 | /mM· msec |
| dissociation constant | $koff_{TnChCa}$ | $3.2 \cdot 10^{-5}$ | / msec |
| association constant | $kon_{TnChMg}$ | 0.003 | /mM· msec |
| dissociation constant | $koff_{TnChMg}$ | 0.00333 | / msec |

| | | | |
|---|---|---|---|
| total $TnC_{high}$ concentration | $Bmax_{TnChigh}$ | 0.14 | mM |
| association constant | $kon_{CaM}$ | 34.0 | /mM· msec |
| dissociation constant | $koff_{CaM}$ | 0.238 | / msec |
| total CaM concentration | $Bmax_{CaM}$ | 0.024 | mM |
| association constant | $kon_{myocinCa}$ | 13.8 | /mM· msec |
| dissociation constant | $koff_{myocinCa}$ | $4.6 \cdot 10^{-4}$ | / msec |
| association constant | $kon_{myocinMg}$ | 0.0157 | /mM· msec |
| dissociation constant | $koff_{myocinMg}$ | $5.7 \cdot 10^{-5}$ | / msec |
| total myocin concentration | $Bmax_{myocin}$ | 0.14 | mM |
| association constant | $kon_{SRB}$ | 100 | /mM· msec |
| dissociation constant | $koff_{SRB}$ | 0.06 | / msec |
| total SRB concentration | $Bmax_{SRB}$ | 0.0171 | mM |

$$\frac{dTnC_{low}}{dt} = kon_{TnClow} \cdot Ca_i \cdot (Bmax_{TnClow} - TnC_{low}) - koff_{TnClow} \cdot TnC_{low}$$

$$\frac{dTnC_{hCa}}{dt} = kon_{TnChCa} \cdot Ca_i \cdot \left(Bmax_{TnChigh} - TnC_{TnChCa} - TnC_{TnChMg}\right) - koff_{TnChCa} \cdot TnC_{TnChCa}$$

$$\frac{dTnC_{hMg}}{dt} = kon_{TnChMg} \cdot Mg_i \cdot \left(Bmax_{TnChigh} - TnC_{TnChCa} - TnC_{TnChMg}\right) - koff_{TnChMg} \cdot TnC_{TnChMg}$$

$$\frac{dCaM}{dt} = kon_{CaM} \cdot Ca_i \cdot (Bmax_{CaM} - CaM) - koff_{CaM} \cdot CaM$$

$$\frac{dMyocin_{Ca}}{dt} = kon_{myocinCa} \cdot Ca_i \cdot \left(Bmax_{myocin} - Myocin_{Ca} - Myocin_{Mg}\right) - koff_{myocinCa} \cdot Myocin_{Ca}$$

$$\frac{dMyocin_{Mg}}{dt} = kon_{myocinMg} \cdot Mg_i \cdot \left(Bmax_{myocin} - Myocin_{Ca} - Myocin_{Mg}\right) - koff_{myocinMg} \cdot Myocin_{Mg}$$

$$\frac{dSRB}{dt} = kon_{SRB} \cdot Ca_i \cdot (Bmax_{SRB} - SRB) - koff_{SRB} \cdot SRB$$

$$JCaB_{total} = \frac{dTnC_{low}}{dt} + \frac{dTnC_{hCa}}{dt} + \frac{dTnC_{hMg}}{dt} + \frac{dCaM}{dt} + \frac{dMyocin_{Ca}}{dt} + \frac{dMyocin_{Mg}}{dt} + \frac{dSRB}{dt}$$

Junctional space $Ca^{2+}$ buffers

| | Abbreviation | Value | Unit |
|---|---|---|---|
| association constant | $kon_{JSlow}$ | 100 | /mM· msec |
| dissociation constant | $koff_{JSlow}$ | 1.3 | / msec |
| total $JS_{low}$ concentration | $Bmax_{JSlow}$ | $3.22 \cdot 10^{-4} \cdot \frac{Vol_{cyt}}{Vol_{JS}}$ | mM |

| | | | |
|---|---|---|---|
| association constant | $kon_{JShigh}$ | 100 | /mM· msec |
| dissociation constant | $koff_{JShigh}$ | 0.03 | / msec |
| total $JS_{high}$ concentration | $Bmax_{JShigh}$ | $1.155 \cdot 10^{-4} \cdot \frac{Vol_{cyt}}{Vol_{JS}}$ | mM |
| $\frac{dJS_{low}}{dt} = kon_{JSlw} \cdot Ca_{JS} \cdot (Bmax_{JSlow} - JS_{low}) - koff_{JSlow} \cdot JS_{low}$ <br> $\frac{dJS_{high}}{dt} = kon_{JShigh} \cdot Ca_{JS} \cdot (Bmax_{JShigh} - JS_{high}) - koff_{JShigh} \cdot JS_{high}$ <br> $JCaB_{JS} = \frac{dJS_{low}}{dt} + \frac{dJS_{high}}{dt}$ | | | |

Subsarcolemmal space $Ca^{2+}$ buffers

| | Abbreviation | Value | Unit |
|---|---|---|---|
| association constant | $kon_{SLlow}$ | 100 | /mM· msec |
| dissociation constant | $koff_{SLlow}$ | 1.3 | / msec |
| total $SL_{low}$ concentration | $Bmax_{SLlow}$ | $0.02618 \cdot \frac{Vol_{cyt}}{Vol_{SL}}$ | mM |
| association constant | $kon_{SLhigh}$ | 100 | /mM· msec |
| dissociation constant | $koff_{SLhigh}$ | 0.03 | / msec |
| total $SL_{high}$ concentration | $Bmax_{SLhigh}$ | $0.00938 \cdot \frac{Vol_{cyt}}{Vol_{SL}}$ | mM |
| $\frac{dSL_{low}}{dt} = kon_{SLlow} \cdot Ca_{SL} \cdot (Bmax_{SLlow} - SL_{low}) - koff_{SLlow} \cdot SL_{low}$ <br> $\frac{dSL_{high}}{dt} = kon_{SLhigh} \cdot Ca_{SL} \cdot (Bmax_{SLhigh} - SL_{high}) - koff_{SLhigh} \cdot SL_{high}$ <br> $JCaB_{SL} = \frac{dSL_{low}}{dt} + \frac{dSL_{high}}{dt}$ | | | |

# $Ca^{2+}$ fluxes;

$J_{cytSL}$; $Ca^{2+}$ diffusion between myoplasm and subsarcolemmal space

| | Abbreviation | Value | Unit |
|---|---|---|---|
| permeability | $P_{cytSL}$ | 5000 | μm³/msec |
| $J_{cytSL} = P_{cytSL} \cdot (Ca_{SL} - Ca_i)$ | | | |

$J_{JS\_SL}$; $Ca^{2+}$ diffusion between junctional space and subsalcolemmal space

| | Abbreviation | Value | Unit |
|---|---|---|---|
| permeability | $P_{JS\_SL}$ | 824.13 | μm³/msec |
| $J_{JS\_SL} = P_{JS\_SL} \cdot (Ca_{SL} - Ca_{JS})$ | | | |

## Table S4. SR $Ca^{2+}$ fluxes

$I_{SERCA}$; SR $Ca^{2+}$ pump current

| | Abbreviation | Value | Unit |
|---|---|---|---|
| maximum velocity | Vmax | 0.0075 | mM/msec |
| dissociation constant | $Km_f$ | $2.0 \cdot 10^{-4}$ | mM |
| dissociation constant | $Km_r$ | 1.7 | mM |

**For MSI model**

$$I_{SERCA} = 0.7 \cdot \frac{Vmax \cdot Vol_{SR} \cdot F \cdot 2}{Cm} \cdot \frac{\left(\left(\frac{Ca_i}{Km_f}\right)^{1.787} - \left(\frac{Ca_{SR}}{Km_r}\right)^{1.787}\right)}{1 + \left(\frac{Ca_i}{Km_f}\right)^{1.787} + \left(\frac{Ca_{SR}}{Km_r}\right)^{1.787}}$$

**For non-MSI model & non-MSI model with high $CU_{mit\_cyt}$ & $NCX_{mit\_cyt}$**

$$I_{SERCA} = 1.0 \cdot \frac{Vmax \cdot Vol_{SR} \cdot F \cdot 2}{Cm} \cdot \frac{\left(\left(\frac{Ca_i}{Km_f}\right)^{1.787} - \left(\frac{Ca_{SR}}{Km_r}\right)^{1.787}\right)}{1 + \left(\frac{Ca_i}{Km_f}\right)^{1.787} + \left(\frac{Ca_{SR}}{Km_r}\right)^{1.787}}$$

$I_{RyR}$; $Ca^{2+}$ current through RyR channel

| | Abbreviation | Value | Unit |
|---|---|---|---|
| | $ec50_{SR}$ | 0.25 | mM |
| | $ko_{Ca}$ | 10.0 | $mM^2$/msec |
| | $ko_m$ | 0.06 | /msec |
| | $ki_{Ca}$ | 0.5 | /mM· msec |
| | $ki_m$ | 0.005 | /msec |
| | ks | 100 | /msec |

$$Max_{SR} = 15$$

$$Min_{SR} = 1$$

$$k_{Ca_{SR}} = Max_{SR} - \frac{Max_{SR} - Min_{SR}}{1 + \left(\frac{ec_{50_{SR}}}{Ca_{SR}}\right)^{2.5}}$$

$$ko_{SR_{Ca}} = \frac{ko_{Ca}}{k_{Ca_{SR}}}$$

$$ki_{SR_{Ca}} = ki_{Ca} \cdot k_{Ca_{SR}}$$

$$RI = 1 - RyR_r - RyR_o - RyR_i$$

$$\frac{dRyR_r}{dt} = \left(ki_m \cdot RI - ki_{SR_{Ca}} \cdot Ca_{JS} \cdot RyR_r\right) - \left(ko_{SR_{Ca}} \cdot Ca_{JS}^2 \cdot RyR_r - ko_m \cdot RyR_o\right)$$

$$\frac{dRyR_o}{dt} = \left(ko_{SR_{Ca}} \cdot Ca_{JS}^2 \cdot RyR_r - ko_m \cdot RyR_o\right) - \left(ki_{SR_{Ca}} \cdot Ca_{JS} \cdot RyR_o - ki_m \cdot RyR_i\right)$$

| |
|---|
| $$\frac{dRyR_i}{dt} = \left(ki_{SR_{Ca}} \cdot Ca_{JS} \cdot RyR_o - ki_m \cdot RyR_i\right) - \left(ko_m \cdot RyR_i - ko_{SR_{Ca}} \cdot Ca_{JS}^2 \cdot RI\right)$$ $$I_{RyR} = k_s \cdot \frac{Vol_{SR} \cdot F \cdot 2}{Cm} \cdot RyR_o \cdot \left(Ca_{SR} - Ca_{JS}\right)$$ |

$I_{leak}$; Leak $Ca^{2+}$ current through SR

| | Abbreviation | Value | Unit |
|---|---|---|---|
| maximum velocity | Vmax | $2.0 \cdot 10^{-5}$ | /msec |
| $$I_{leak} = V_{max} \cdot \frac{Vol_{cyt} \cdot F \cdot 2}{Cm} \cdot \left(Ca_{SR} - Ca_{JS}\right)$$ | | | |

Calsequestrin; $Ca^{2+}$ buffer in SR

| | Abbreviation | Value | Unit |
|---|---|---|---|
| association constant | $kon_{csqn}$ | 100 | /mM· msec |
| dissociation constant | $koff_{csqn}$ | 65.0 | / msec |
| total calsequestrin concentration | $Bmax_{csqn}$ | $0.13 \cdot \frac{Vol_{cyt}}{Vol_{SR}}$ | mM |
| $$\frac{dCsqn_b}{dt} = kon_{csqn} \cdot Ca_{SR} \cdot \left(Bmax_{csqn} - Csqn_b\right) - koff_{csqn} \cdot Csqn_b$$ | | | |

# Table S5. Mitochondrial $Ca^{2+}$ fluxes

$I_{CUmit}$; Mitochondrial $Ca^{2+}$ uniporter current

| | Abbreviation | Value | Unit |
|---|---|---|---|
| | $\alpha_{mit}$ | 0.2 | |
| | $\alpha_i$ | 0.341 | |
| | $Ki_{Camit}$ | 0.01 | mM |
| amplitude | $A_{CaUni}$ | 185.6 | L/msec |
| **For MSI model** $$I_{CU_{mit\_cyt}} = -A_{CaUni} \cdot \frac{F \cdot 2}{Cm} \cdot \left(2 \cdot \Delta\psi \cdot \frac{F}{RT}\right) \cdot \frac{\alpha_{mit} \cdot Ca_{mit} - \alpha_i \cdot Ca_i \cdot \exp\left(-2 \cdot \Delta\psi \cdot \frac{F}{RT}\right)}{\exp\left(-2 \cdot \Delta\psi \cdot \frac{F}{RT}\right) - 1} \cdot \frac{(Ki_{Camit})^2}{(Ca_{mit})^2 + (Ki_{Camit})^2}$$ | | | |

$$I_{CU_{mit\_JS}} = -\mathrm{A}_{\mathrm{CaUni}} \cdot \frac{F \cdot 2}{Cm} \cdot \left(2 \cdot \Delta\psi \cdot \frac{F}{RT}\right) \cdot \frac{\alpha_{mit} \cdot Ca_{mit} - \alpha_i \cdot Ca_{JS} \cdot \exp\left(-2 \cdot \Delta\psi \cdot \frac{F}{RT}\right)}{\exp\left(-2 \cdot \Delta\psi \cdot \frac{F}{RT}\right) - 1} \cdot \frac{(Ki_{Camit})^2}{(Ca_{mit})^2 + (Ki_{Camit})^2}$$

**For non-MSI model**

$$I_{CU_{mit\_cyt}} = -\mathrm{A}_{\mathrm{CaUni}} \cdot \frac{F \cdot 2}{Cm} \cdot \left(2 \cdot \Delta\psi \cdot \frac{F}{RT}\right) \cdot \frac{\alpha_{mit} \cdot Ca_{mit} - \alpha_i \cdot Ca_i \cdot \exp\left(-2 \cdot \Delta\psi \cdot \frac{F}{RT}\right)}{\exp\left(-2 \cdot \Delta\psi \cdot \frac{F}{RT}\right) - 1} \cdot \frac{(Ki_{Camit})^2}{(Ca_{mit})^2 + (Ki_{Camit})^2}$$

**For non-MSI model with high $CU_{mit\_cyt}$ & $NCX_{mit\_cyt}$**

$$I_{CU_{mit\_cyt}} = -2 \cdot \mathrm{A}_{\mathrm{CaUni}} \cdot \frac{F \cdot 2}{Cm} \cdot \left(2 \cdot \Delta\psi \cdot \frac{F}{RT}\right) \cdot \frac{\alpha_{mit} \cdot Ca_{mit} - \alpha_i \cdot Ca_i \cdot \exp\left(-2 \cdot \Delta\psi \cdot \frac{F}{RT}\right)}{\exp\left(-2 \cdot \Delta\psi \cdot \frac{F}{RT}\right) - 1} \cdot \frac{(Ki_{Camit})^2}{(Ca_{mit})^2 + (Ki_{Camit})^2}$$

$I_{NCXmi\_cyt}$; Current of mitochondrial $Na^+$-$Ca^{2+}$ exchanger facing cytoplasm

| | Abbreviation | Value | Unit |
|---|---|---|---|
| dissociation constants | $KdNa_{mit}$ | 38.0 | mM |
| | $KdNa_i$ | 32.0 | mM |
| | $KdCa_{mit}$ | 0.020932007 | mM |
| | $KdCa_i$ | 0.0125 | mM |
| amplitude | $A_{NCXmit}$ | 21.0192 | mmol/msec |

$$PNa_{mit} = \frac{Na_{mit}^3}{Na_{mit}^3 + Kd_{Namit}^3 \cdot \left(1 + \frac{Ca_{mit}}{KdCa_{mit}}\right)}$$

$$PCa_{mit} = \frac{Ca_{mit}}{Ca_{mit} + KdCa_{mit} \cdot \left(1 + \frac{Na_{mit}^3}{Kd_{Namit}^3}\right)}$$

$$PNa_i = \frac{Na_i^3}{Na_i^3 + Kd_{Nai}^3 \cdot \left(1 + \frac{Ca_i}{KdCa_i}\right)}$$

$$PCa_i = \frac{Ca_i}{Ca_i + KdCa_i \cdot \left(1 + \frac{Na_i^3}{Kd_{Nai}^3}\right)}$$

$$k1 = 1.0 \cdot \exp\left(0.2 \cdot F \cdot \Delta\psi \cdot \frac{F}{RT}\right) \cdot PNa_{mit}$$

$$k2 = 1.0 \cdot \exp\left((0.2 - 1.0) \cdot F \cdot \Delta\psi \cdot \frac{F}{RT}\right) \cdot PNa_{i}$$

$$k3 = 1.0 \cdot PCa_{mit}$$

$$k4 = 1.0 \cdot PCa_{i}$$

$$tE1 = \frac{\alpha}{\alpha + \beta}$$

$tE2 = 1 - tE1$ where $\alpha = (k2+k4)$, $\beta = (k1+k3)$

**For MSI model and non-MSI model**

$$I_{NCX_{mit\_cyt}} = -A_{NCX_{mit}} \cdot \frac{F \cdot 2}{Cm} \cdot (tE1 \cdot k2 - tE1 \cdot k1)$$

**For non-MSI model with high $CU_{mit\_cyt}$ & $NCX_{mit\_cyt}$**

$$I_{NCX_{mit\_cyt}} = -\left(1 + \frac{7}{3}\right) \cdot A_{NCX_{mit}} \cdot \frac{F \cdot 2}{Cm} \cdot (tE1 \cdot k2 - tE1 \cdot k1)$$

$I_{NmSC}$; NCLX–SERCA complex current

| | Abbreviation | Value | Unit |
|---|---|---|---|
| dissociation constants | $KdNa_{mit}$ | 38.0 | mM |
| | $KdNa_{i}$ | 32.0 | mM |
| | $KdCa_{mit}$ | 0.020932007 | mM |
| | $KdCa_{i}$ | 0.0125 | mM |
| amplitude | $A_{NmSC}$ | 49.0448 | mmol/msec |

$$PNa_{mit} = \frac{Na_{mit}^3}{Na_{mit}^3 + Kd_{Namit}^3 \cdot \left(1 + \frac{Ca_{mit}}{KdCa_{mit}}\right)}$$

$$PCa_{mit} = \frac{Ca_{mit}}{Ca_{mit} + KdCa_{mit} \cdot \left(1 + \frac{Na_{mit}^3}{Kd_{Namit}^3}\right)}$$

$$PNa_{i} = \frac{Na_{i}^3}{Na_{i}^3 + Kd_{Nai}^3 \cdot \left(1 + \frac{Ca_{i}}{KdCa_{i}}\right)}$$

$$PCa_{i} = \frac{Ca_{i}}{Ca_{i} + KdCa_{i} \cdot \left(1 + \frac{Na_{i}^3}{Kd_{Nai}^3}\right)}$$

$$k1 = 1.0 \cdot \exp\left(0.2 \cdot F \cdot \Delta\psi \cdot \frac{F}{RT}\right) \cdot PNa_{mit}$$

$$k2 = 1.0 \cdot \exp\left((0.2 - 1.0) \cdot F \cdot \Delta\psi \cdot \frac{F}{RT}\right) \cdot PNa_i$$

$$k3 = 1.0 \cdot PCa_{mit}$$

$$k4 = 1.0 \cdot PCa_i$$

$$tE1 = \frac{\alpha}{\alpha + \beta}$$

$tE2 = 1 - tE1$ where α = ($k2$+$k4$), β = ($k1$+$k3$)

$$I_{NmSC} = -A_{NmSC} \cdot \frac{F \cdot 2}{Cm} \cdot (tE1 \cdot k2 - tE1 \cdot k1)$$

$Ca^{2+}$ buffer in mitochondria

| | Abbreviation | Value | Unit |
|---|---|---|---|
| association constant | $kon_{mit}$ | 100 | /mM· msec |
| dissociation constant | $koff_{mit}$ | 0.1 | / msec |
| total calsequestrin concentration | $Bmax_{mit}$ | 0.6 | mM |

$$\frac{dCaBuff_{mit}}{dt} = kon_{mit} \cdot Ca_{mit} \cdot (Bmax_{mit} - CaBuff_{mit}) - koff_{mit} \cdot CaBuff_{mit}$$

## Table S6. Nernst potentials

$$E_{Na} = \frac{RT}{F} \ln \frac{Na_o}{Na_i}$$

$$E_K = \frac{RT}{F} \ln \frac{K_o}{K_i}$$

$$E_{CaJS} = \frac{RT}{F} \ln \frac{Ca_o}{Ca_{JS}}$$

$$E_{CaSL} = \frac{RT}{F} \ln \frac{Ca_o}{Ca_{SL}}$$

$$E_{Cl} = -\frac{RT}{F} \ln \frac{Cl_o}{Cl_i}$$

## Table S7. Ion concentrations

$Na^+$ concentrations

$$INa_{totJS} = INa_{JS} + 3 \cdot INaK_{JS} + 3 \cdot INaCa_{JS} + ICaL_{NaJS} + Iha_{NaJS}$$

$$INa_{totSL} = INa_{SL} + 3 \cdot INaK_{SL} + 3 \cdot INaCa_{SL} + ICaL_{NaSL} + Iha_{NaSL}$$

$$INa_{tot} = INa_{totJS} + INa_{totSL}$$

$$\frac{dNa_i}{dt} = 0$$

$K^+$ concentrations

$$IK_{totJS} = IKr_{JS} + IKs_{JS} + IKp_{JS} + Ito_{JS} + IKur_{JS} + IK1_{JS} + ICaL_{KJS} + Iha_{KJS} - 2 \cdot INaK_{JS}$$

$$IK_{totSL} = IKr_{SL} + IKs_{SL} + IKp_{SL} + Ito_{SL} + IKur_{SL} + IK1_{SL} + ICaL_{KSL} + Iha_{KSL} - 2 \cdot INaK_{SL}$$

$$IK_{tot} = IK_{totJS} + IK_{totSL}$$

$$\frac{dK_i}{dt} = 0$$

$Cl^-$ concentrations

$$ICl_{totJS} = IClCa_{JS} + IClb_{JS}$$

$$ICl_{totSL} = IClCa_{SL} + IClb_{SL}$$

$$ICl_{tot} = ICl_{totJS} + ICl_{totSL}$$

$$\frac{dCl_i}{dt} = 0$$

$Ca^{2+}$ concentrations

**For MSI model**

$$ICa_{totJS} = ICaL_{CaJS} + ICaT_{JS} + IpCa_{JS} + ICab_{JS} - 2 \cdot INaCa_{JS}$$

$$ICa_{totSL} = ICaL_{CaSL} + ICaT_{SL} + IpCa_{SL} + ICab_{SL} - 2 \cdot INaCa_{SL}$$

$$ICa_{tot} = ICa_{totJS} + ICa_{totSL}$$

$$\frac{dCa_{js}}{dt} = -\left(ICa_{totJS} - ICU_{mit\_JS} - IRyR - Ileak\right) \cdot \frac{Cm}{Vol_{JS} \cdot 2 \cdot F} + \frac{J_{JS\_SL}}{Vol_{JS}} - JCaB_{JS}$$

$$\frac{dCa_{SL}}{dt} = -ICa_{totSL} \cdot \frac{Cm}{Vol_{SL} \cdot 2 \cdot F} - \frac{J_{JS\_SL}}{Vol_{SL}} - \frac{J_{cytSL}}{Vol_{SL}} - JCaB_{SL}$$

$$\frac{dCa_i}{dt} = -\left(ISERCA - ICU_{mit\_cyt} + 2 \cdot INCX_{mit\_cyt}\right) \cdot \frac{Cm}{Vol_{cyt} \cdot 2 \cdot F} - JCaB_{total} + \frac{J_{cytSL}}{Vol_{cyt}}$$

$$\frac{dCa_{SR}}{dt} = -(-ISERCA + 2 \cdot INmSC + IRyR + Ileak) \cdot \frac{Cm}{Vol_{SR} \cdot 2 \cdot F} - \frac{dCsqn_b}{dt}$$

$$\frac{dCa_{totalmit}}{dt} = -\left(ICU_{mit_{cyt}} + ICU_{mit_{JS}} - 2 \cdot INCX_{mit_{cyt}} - 2 \cdot INmSC\right) \cdot \frac{Cm}{Vol_{mit} \cdot 2 \cdot F} - \frac{dCaBuff_{mit}}{dt}$$

**For non-MSI model**

$$ICa_{totJS} = ICaL_{CaJS} + ICaT_{JS} + IpCa_{JS} + ICab_{JS} - 2 \cdot INaCa_{JS}$$

$$ICa_{totSL} = ICaL_{CaSL} + ICaT_{SL} + IpCa_{SL} + ICab_{SL} - 2 \cdot INaCa_{SL}$$

$$ICa_{tot} = ICa_{totJS} + ICa_{totSL}$$

$$\frac{dCa_{js}}{dt} = -\left(ICa_{totJS} - IRyR - Ileak\right) \cdot \frac{Cm}{Vol_{JS} \cdot 2 \cdot F} + \frac{J_{JS\_SL}}{Vol_{JS}} - JCaB_{JS}$$

$$\frac{dCa_{SL}}{dt} = -ICa_{totSL} \cdot \frac{Cm}{Vol_{SL} \cdot 2 \cdot F} - \frac{J_{JS\_SL}}{Vol_{SL}} - \frac{J_{cytSL}}{Vol_{SL}} - JCaB_{SL}$$

$$\frac{dCa_i}{dt} = -\left(ISERCA - ICU_{mit\_cyt} + 2 \cdot INCX_{mit\_cyt}\right) \cdot \frac{Cm}{Vol_{cyt} \cdot 2 \cdot F} - JCaB_{total} + \frac{J_{cytSL}}{Vol_{cyt}}$$

$$\frac{dCa_{SR}}{dt} = -(-ISERCA + IRyR + Ileak) \cdot \frac{Cm}{Vol_{SR} \cdot 2 \cdot F} - \frac{dCsqn_b}{dt}$$

$$\frac{dCa_{totalmit}}{dt} = -\left(ICU_{mit_{cyt}} - 2 \cdot INCX_{mit_{cyt}}\right) \cdot \frac{Cm}{Vol_{mit} \cdot 2 \cdot F} - \frac{dCaBuff_{mit}}{dt}$$

## Table S8. Membrane potential

$$I_{tot} = INa_{tot} + IK_{tot} + ICa_{tot} + ICl_{tot}$$

$$\frac{dVm}{dt} = -I_{tot}$$
